# Which Intervention Characteristics are Related to More Exposure to Internet-Delivered Healthy Lifestyle Promotion Interventions? A Systematic Review

**DOI:** 10.2196/jmir.1639

**Published:** 2011-01-06

**Authors:** Wendy Brouwer, Willemieke Kroeze, Rik Crutzen, Jascha de Nooijer, Nanne K de Vries, Johannes Brug, Anke Oenema

**Affiliations:** ^4^Department of Epidemiology & Biostatistics and the EMGO+ Institute for Health and Care ResearchVU University Medical CenterAmsterdamNetherlands; ^3^Department of Health Promotion and CAPHRI School for Public Health and Primary CareMaastricht UniversityMaastrichtNetherlands; ^2^Department of Health Sciences and the EMGO+ Institute for Health and Care ResearchVU University AmsterdamAmsterdamNetherlands; ^1^Department of Public HealthErasmus MCUniversity Medical Center RotterdamRotterdamNetherlands

**Keywords:** Systematic review, Internet, Internet intervention, exposure, behavior change

## Abstract

**Background:**

The Internet has become a popular medium for the delivery of tailored healthy lifestyle promoting interventions. The actual reach of Internet-delivered interventions seems, however, lower than expected, and attrition from interventions is generally high. Characteristics of an intervention, such as personally tailored feedback and goal setting, are thought to be among the important factors related to of use of and exposure to interventions. However, there is no systematic overview of which characteristics of Internet-delivered interventions may be related to more exposure.

**Objective:**

The present study aims to identify (1) which potentially exposure-promoting methods and strategies are used in existing Internet interventions, (2) which objective outcome measures are used to measure exposure to Internet interventions, and (3) which potentially exposure-promoting methods and strategies are associated with better exposure.

**Methods:**

A systematic review of the literature was conducted based on the Cochrane guidelines. Papers published between 1995 and 2009 were searched in the PubMed, PsycINFO, and Web of Science databases. In total, 64 studies were included that reported objective exposure measures such as completion of an initial visit, number of log-ins, and time spent on the website. Information about intervention-related characteristics (ie, interactive behavior change strategies, interactive elements for fun, peer or counsel support, email/phone contact, and regular updates of the website) that could potentially contribute to better exposure and objective exposure outcomes were abstracted from the studies and qualitative systematic descriptive analyses were performed.

**Results:**

The results showed that a large variety of behavior change techniques and other exposure-promoting elements were used in the interventions and that these methods and strategies varied for the various lifestyle behaviors. Feedback, interactive elements, and email/phone contact were used most often. In addition, there was much variety and a lack of consistency in the exposure measures that were reported. Of all the categories of intervention characteristics that may be associated with better exposure, there were indications that peer and counselor support result in a longer website visit and that email/phone contact and updates of the website result in more log-ins.

**Conclusions:**

Results of this qualitative systematic review indicate that of all intervention characteristics that could potentially enhance exposure, only peer support, counselor support, email/phone contact with visitors, and updates of the intervention website were related to better exposure. The diversity of intervention methods used and the inconsistency in the report of exposure measures prevented us from drawing firmer conclusions. More research is needed to identify whether other characteristics of Internet interventions are associated with greater exposure.

## Introduction

The Internet has become a primary source for obtaining health information by the public [1-3] making it an interesting medium for providing interventions aimed at promoting healthful behaviors. In the last decade, the number of behavior change interventions that have become available through the Internet has greatly expanded. An advantage of using the Internet as a channel for delivery is the opportunity for health professionals to provide interactive, individualized interventions to large numbers of people [4-8] that match each visitor's unique characteristics, circumstances, beliefs, motivation to change, and behavior [5,9]. Furthermore, a large part of the population can potentially be reached since so many people now have Internet access [10]. The Netherlands is one of the countries with the highest Internet penetration rates, together with Australia, the United States, the United Kingdom, and the Scandinavian countries [10]. Further advantages of the Internet are the easy and constant accessibility of interventions; visitors can access the intervention program at any time and location, can work through the program at their own pace, and can be more anonymous than in face-to-face contacts.

The evidence for efficacy of Internet interventions indicates that Internet-delivered interventions can be effective in changing behaviors even though effect sizes are mostly small [11-15]. However, earlier efficacy studies have indicated that the use of and exposure to the content of Internet interventions may often not be optimal [7,16-18]. Furthermore, visitor engagement in Internet interventions has been found to be lower than initially intended [19], that is, visitors tend to leave the intervention website before completing it [19-21]. This hampers them from being optimally exposed to the intervention content. Many Internet interventions consist of multiple visits, and there is growing evidence that repeated website visits are necessary to achieve sustainable changes [22-24]. Vandelanotte et al [13], for example, reported in a review that better outcome measures regarding improvement of physical activity were identified when participants visited the intervention website more than 5 times. However, other studies reported that only a minority of participants visited an intervention more than once [4,23].

These findings indicate that large improvements can be made with regard to exposure to Internet-delivered interventions, which may contribute to improved intervention efficacy and improved overall impact of an intervention. According to the diffusion of innovations theory [25], characteristics of an innovation (eg, an Internet-delivered intervention) are important in the process of implementation and adoption of an intervention, next to characteristics of users, such as personal characteristics and individual cognitions. In previous—mainly qualitative— studies, a number of intervention-related characteristics have been indicated as potential exposure-enhancing factors [26-31]. Interactive behavior change strategies, such as the provision of individualized computer-tailored feedback and goal setting, may enhance engagement in the intervention content and completion of the program [26-28,31]. Furthermore, intervention elements that make the intervention more attractive to use, such as quizzes, small movies, and other multimedia features, may enhance an extended stay on the website [26,28]. In addition, social support by peers and professionals may enhance an extended stay on the website and may encourage a revisit to an intervention website [26-28,31]. Furthermore, the possibility to monitor progress toward behavior change, the provision of regular new content, and periodic prompts and reminders may improve revisits [26-31]. Even though there is some evidence for intervention characteristics that may enhance exposure, there is no systematic overview of which intervention characteristics are associated with more exposure to Internet interventions. With respect to objective exposure measures, various relevant exposure measures have been suggested in previous studies [4,32], such as accessing the intervention content, number of modules or sessions completed during single or multiple visits, webpage viewing, visit duration, frequency of website visits, and use of specific elements in the intervention (eg, use of self-monitoring tool or bulletin board). The aim of the present study was to conduct a systematic review of the literature and to provide an overview of which characteristics of an intervention are related to better use of and exposure to an Internet intervention. Three specific research questions guided our systematic review: (1) Which potentially exposure-promoting methods and strategies are used in existing Internet interventions? (2) Which objective outcome measures are used to measure exposure to Internet interventions? (3) Which potentially exposure-promoting methods and strategies are associated with better exposure?

## Methods

The review was conducted using a review protocol that was developed based on the Cochrane guidelines for systematic reviews [33].

### Search Strategy

A structured electronic database search of PubMed, PsycINFO, and Web of Science was conducted for Internet intervention studies published from January 1, 1995, through February 8, 2009. The following search terms were used: "Internet" or "Web" or "online" and "health promotion" or "health education" or "health communication" or "health planning" or "prevention" or "intervention" or "behavio* change" or "behavio* modification." The search was limited to the interventions among adults (18 years and older) and English-language peer-reviewed publications. This search strategy was optimized for all consulted databases.

### Inclusion and Exclusion Criteria

A study was eligible for inclusion if it described an Internet intervention that aimed at the primary prevention of physical chronic diseases among the general public from the age of 18. Relevant behaviors included physical activity, nutrition, weight management, smoking cessation, alcohol consumption, or a combination of these behaviors. Furthermore, the Internet interventions needed to be developed for use among the general public. Next, objective quantitative exposure measures (eg, number of log-ins, number of pages visited, completion of the entire intervention or parts of the intervention, time spent on the intervention website, number of visits to the intervention) needed to be reported. Finally, studies evaluating an intervention in a randomized controlled trial (RCT), a quasi-experimental design, or describing use of an intervention only in a single group study could be included.

### Review Procedure

The selection of studies took place in 3 phases based on title (author WB), abstract (authors WB and WK), and full publication (WB and WK). Title and abstract screening were done blinded for author, journal, and date of publication. If in doubt about suitability of a study in one phase, the study was included in the next phase. Disagreements on inclusion in the third phase were discussed with a third reviewer (author AO) until consensus was reached.

### Data Extraction and Analysis

Data from the included studies were extracted by a team of reviewers and then verified and tabulated for this review by WB, WK, and AO. Based on a standardized extraction form, descriptive key elements and objective exposure measures of all included studies were summarized and presented in tables (Multimedia Appendix 1 and Multimedia Appendix 2). For this extraction, we relied on the information about the study and intervention provided in the published literature (ie, the selected publication, publications that evaluated and reported on the same intervention [see Table 1 for applicable studies], and references to additional design papers or appendices).

Potential exposure-increasing methods and strategies that have been found to be effective in previous studies were divided into the following categories: (1) interactive behavior change strategies, which include methods and strategies delivered in an interactive format (eg, tailored feedback, goal setting tools, action planning tools, or self-monitoring tools); (2) interactive elements, which include elements of the program that are more for fun to improve the attractiveness of the intervention or to provide the option for more information (eg, quizzes, searchable databases, or audio/video); (3) peer support (eg, forum, bulletin board, or chat); (4) counselor support (eg, ask-the-expert, email/phone contact, or counselor-led chat sessions); (5) email and/or phone contact, which may include email/phone messages providing intervention content (eg, personal feedback or newsletters) or email/phone prompts to remind users to revisit the intervention; (6) update of the information on the intervention website, which include, for example, new tools, information, or news; (7) intervention incentives, which refer to incentives that are related to using the Internet intervention and not related to taking part in a study.

For consistency and comparability among studies, the taxonomy of Abraham and Michie was used for the description of interactive behavior change strategies [34]. Within computer-tailored feedback, various types of feedback can be distinguished, such as feedback on performance, cognitive constructs, barrier identification and solutions, and cognitive and behavioral processes. In this study, we considered tailored feedback as one interactive behavior change strategy. Feedback on progress was included separately as this kind of feedback can only be given during a revisit.

Due to the significant heterogeneity between the studies and the variation in the reported exposure measures, the data could not be pooled for quantitative analysis. Therefore, qualitative, systematic, descriptive analyses were performed. This method has been proven to be suitable for systematic reviews [35].

To gain insight into which intervention characteristics may result in better exposure, the studies were listed in a matrix, linking the potential exposure-promoting intervention elements with the outcome measures (Tables 3 and 4). The objective exposure measures used in the different studies were very diverse and presented in different statistics (see Multimedia Appendix 1). Therefore, only those objective exposure measures that are used frequently and presented in the same statistic value are presented in Tables 3 and 4. In Tables 3 and 4, a division was made between interventions that offered fewer than 3 versus more than or equal to 3 interactive behavior change strategies, and that offered interactive elements (yes vs no), peer support (yes vs no), counselor support (yes vs no), email/phone contact (yes vs no), update of the intervention website (yes vs no), and intervention incentive (yes vs no). From this matrix, patterns could emerge indicating that the existence of certain intervention characteristics could result in more exposure to the intervention. Criteria for determining that an exposure-promoting element is probably related to an exposure outcome were that at least 50% of the Internet interventions that included the specific exposure-promoting element should be in the highest exposure category and that the number of studies in the highest category differed substantially (at least 35% difference) from the number of interventions without that element in the highest category of exposure. Only when there was a good balance in the number of interventions that did or did not have a specific exposure-promoting element, inferences about a relation between exposure promoting elements and exposure could be made.

## Results

### Study Selection

**Figure 1:**
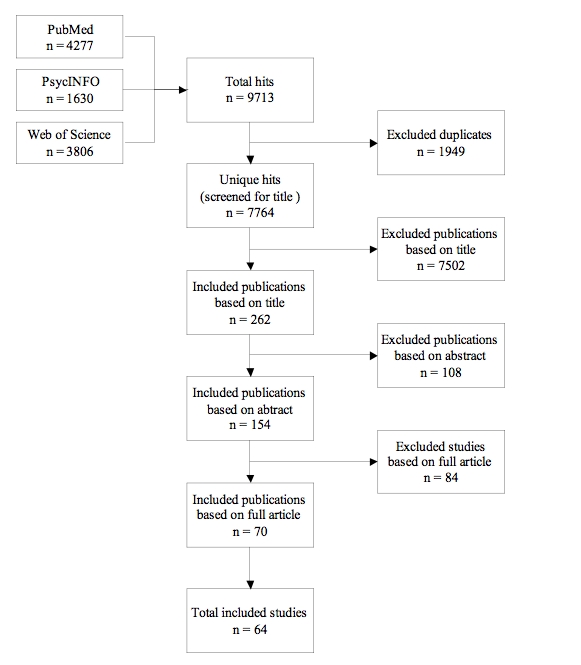
Flow chart review procedure

The initial cross-database search yielded 7764 unique publications (Figure 1). After reviewing titles, abstracts, and full publications, 70 publications describing 64 studies were eligible for inclusion in the review (see Table 1). In total, 192 publications were excluded based on abstract and full publication. The most common reason for exclusion in this phase was that a publication did not describe an Internet intervention aimed at the primary prevention of physical chronic diseases (n = 112). Other publications were excluded because they focused on persons below the age of 18 (n = 11), were not targeted at the general public as end users (n = 3), or did not describe the evaluation of an Internet intervention (n = 37). Finally, 29 publications were excluded, as they did not include objective exposure outcome measures.

**Table 1:** List of included publications for review by behavior (see Multimedia Appendix 1 and Multimedia Appendix 2 for details on study characteristics and intervention descriptions)

**A. Physical activity**	
	1. Carr et al [36], 2008, United States
	2. Dunton and Robertson [37], 2008, United States
	3. Ferney et al [28], 2008, Australia
	4. Herman et al [38], 2006, United States
	5. Hurling et al [39], 2007, United Kingdom
	6. Hurling et al [40], 2006, United Kingdom
	7. Lewis et al [41], 2008; Marcus et al [42], 2007, United States
	8. Leslie et al [16], 2005, Australia
	9. Plotnikoff et al [43], 2006, Canada
	10. Spittaels and De Bourdeaudhuij [44], 2006, Belgium
	11. Spittaels et al [45], 2007, Belgium
	12. Steele et al [46,47], 2007, Australia
**B. Nutrition**	
	13. Buller et al [48], 2008; Woodall et al [49], 2007, United States
	14. Huang et al [50], 2006, Australia
	15. McNeill et al [51], 2007, United States
	16. Papadaki and Scott [52], 2005; Papadaki and Scott [53], 2006, Scotland
**C. Weight management**	
	17. Cussler et al [54], 2008, United States
	18. Glasgow et al [21], 2007, United States
	19. Gold et al [55], 2007, United States
	20. Harvey-Berino et al [56], 2002, United States
	21. Hunter et al [57], 2008, United States
	22. McConnon et al [58], 2007, United Kingdom
	23. McCoy et al [59], 2005, Australia
	24. Micco et al [60], 2007, United States^b^
	25. Petersen et al [61], 2008, United States
	26. Tate et al [62], 2001, United States
	27. Tate et al [63], 2006, United States
	28. Webber et al [64], 2008, United States
	29. Van Wier et al [65], 2009, Netherlands
	30. Wing et al [66], 2006, United States
**D. Smoking cessation**	
	31. Balmford et al [67], 2008, Australia
	32. Brendryen et al [68], 2008, Norway
	33. Brendryen and Kraft [69], 2008, Norway
	34. Cobb et al [70], 2005, United States
	35. Danaher et al [32], 2006, United States
	36. Feil et al [71], 2003, United States
	37. Graham et al [72], 2007, United States
	38. Houston and Ford [73], 2008, United States
	39. Lenert et al [22], 2003, United States
	40. McKay et al [74], 2008, United States
	41. Saul et al [75], 2007, United States
	42. Severson et al [31], 2008, United States
	43. Stoddard et al [76], 2005, United States
	44. Stoddard et al [77], 2008, United States
	45. Strecher et al [78], 2005, England and Ireland
	46. Strecher et al [79,80], 2008, United States
	47. Swartz et al [81], 2006, United States
	48. Wang and Etter [82], 2004, Switzerland
**E. Alcohol reduction**	
	49. Cloud and Peacock [83], 2001, United States
	50. Cunningham et al [84], 2000, Canada
	51. Lieberman [85], 2006, United States
	52. Linke et al [86], 2004; Linke et al [87], 2005, United Kingdom
	53. Linke et al [88], 2007, United Kingdom
	54. Matano et al [89], 2007, United States
	55. Riper et al [90], 2008, Netherlands
	56. Saitz et al [91], 2004, United States
	57. Westrup et al [92], 2003, United States
**F. Combination of behaviors**	
	58. Cook et al [93], 2007, United States
	59. Cowdery et al [94], 2007, United States
	60. Oenema et al [95], 2008, Netherlands
	61. Verheijden et al [23], 2007, Netherlands
	62. Ware et al [96], 2008, United Kingdom
	63. Winett et al [97], 2007, United States
	64. Woolf et al [98], 2006, United States

### Characteristics of Selected Studies

Of the 64 included studies, 39 were performed in the United States, 6 in Australia, 6 in the United Kingdom and Ireland, 4 in the Netherlands, 2 in Belgium, 2 in Canada, 2 in Norway, and 1 in Switzerland. In all, 12 studies described in 14 publications (hereafter, the number of publications referenced may exceed the number of studies to which they refer) targeted physical activity [16,28,36-47], 4 targeted nutrition (eg fruit, vegetable, or saturated fat consumption) [48-53], 14 targeted weight management (eg, weight loss/reduction or weight maintenance/control) [21,54-66], 18 targeted smoking cessation [22,31,32,67-82], 9 targeted alcohol reduction [83-92], and 7 targeted multiple behaviors [23,93-98]. Most studies had an RCT design and 14 studies were observational one-group studies evaluating use of the Internet intervention. The length of the interventions varied from a one-time visit to 18 months with multiple visits. The majority of the Internet interventions were explicitly informed by one or more behavioral theories. The social cognitive theory [99], the transtheoretical model in total [100], or the stages of change concept from this model only [100] were used most often. A more detailed description of the study characteristics can be found Multimedia Appendix 2.

### Characteristics of Study Populations

Multimedia Appendix 2 shows that the number of study participants ranged from 32 to 67,324 with an overall mean of 3367 participants and a median of 408. The mean age varied from 32 to 52 years and the percentage of female participants ranging from 2% to 100%. The percentage of participants with education at a level higher than high school (if reported) varied from 41% to 100%.

### Exposure-Improving Methods and Strategies

Table 2 lists the potential exposure-improving methods and strategies used in the interventions. If two or more Internet interventions were described in one publication, the most extended intervention or the intervention that delivered the content mostly through the Internet is taken into account. 

**Table 2:** Potential exposure-improving methods and strategies applied in the Internet interventions for the various health-related behaviors (see Multimedia Appendix 1 for details)

		Physical Activity (n 12)		Nutrition (n = 4)		Weight Management (n = 14)		Smoking Cessation (n = 18)		Alcohol Consumption (n = 9)		Multiple Behaviors (n = 7)		Total (N = 64)	
		n	%	n	%	n	%	n	%	n	%	n	%	n	%
**Interactive Behavior Change Strategy**															
	Feedback^a^	8	67	1	25	7	50	15	83	9	100	7	100	47	73
	Goal setting	7	58	0	0	5	36	0	0	1	11	3	43	16	25
	Action/activity planning	8	67	0	0	2	14	12	67	0	0	3	43	25	39
	Self-monitoring	8	67	0	0	11	79	6	33	5	56	3	43	33	52
	Feedback on progress	7	58	0	0	6	43	2	11	3	33	5	72	23	36
Interactive elements^b^		9	75	4	100	8	57	10	56	8	89	5	72	44	69
Peer support		5	42	1	25	9	64	10	56	5	56	1	14	31	48
Counselor support		4	33	1	25	10	71	9	50	0	0	0	0	24	38
Email/phone contact		9	75	3	75	12	86	14	78	2	22	3	43	43	67
Update		5	42	2	50	8	57	6	33	3	33	2	29	26	41
Intervention incentive		2	17	2	50	6	43	1	6	0	0	0	0	11	17

^a^ Feedback includes feedback on performance, cognitive constructs, barrier identification and solutions, and cognitive and behavioral processes.

^b^ Interactive elements are, for example, quizzes, searchable databases or libraries, heart rate/BMI calculator, and website links.

Multimedia Appendix 1 provides a more detailed description of the methods and strategies applied in each Internet intervention.

The provision of tailored feedback (eg, on performance, cognitive constructs, barrier identification and solutions, and cognitive and behavioral processes) was the most often used behavior change strategy across the behaviors except for nutrition and weight management interventions. Goal setting was offered more often in physical activity interventions; action/activity planning was most often used in the physical activity and smoking cessation interventions and self-monitoring in the physical activity and weight management interventions. Feedback on progress was most often used in the multiple behavior interventions, followed by physical activity. The majority of the interventions in all behavioral domains included interactive elements such as quizzes, searchable databases or libraries, heart rate/BMI calculator, and website links, with less use of these elements in weight management and smoking cessation interventions. Peer support was most often used in the weight management, smoking cessation, and alcohol consumption interventions, while counselor support was most common in the weight management interventions, followed by the smoking cessation interventions. Email/phone contact was frequently used in most interventions except for the alcohol consumption and multiple behavior interventions. Regular updates of the intervention website or provision of an incentive for using the intervention were not often used, but when they were, they were used most in the weight management, nutrition and PA interventions.

### Objective Exposure Outcome Measures

A large variety of exposure measures were used in the included studies (see Multimedia Appendix 1). The frequency of visits by means of log-in rates was the most commonly used exposure outcome measure (n = 33) although the way in which the data were presented was not consistent across studies as different statistics were used (eg, mean or median). There were also several studies that did not present log-in rates but did present the percentage of users that revisited the intervention (n = 9). Other often used outcome measures were how many people landed on the website, which was mostly registered by “hits” on the website (n = 10), the number of visitors that accessed the program content (n = 24), the number of pages visited (n = 6), completion of the first visit or module (n = 13), and completion of the whole intervention (n = 8). Furthermore, use of intervention methods and/or strategies were also presented as exposure measures, such as use of specific intervention components (interactive behavior change strategies and interactive elements [n = 26], use of peer support [n = 12], and use of counselor support [n = 10]).

### Combining Outcome Measures With Potential Exposure-Promoting Methods and Strategies

In Tables 3 and 4, the studies are listed in matrices combining the objective outcome measures that were mostly presented and the potential exposure-promoting elements. Of all the potential exposure-promoting elements listed in Tables 3 and 4, indications were found for peer support, counselor support, email and/or phone contact with visitors, and updates of the intervention website to be related with more exposure. The provision of peer and counselor support appears to have had a positive influence on the time visitors spent on the website. This can be deduced from the finding that at least 50% of the studies evaluating interventions that included peer or counselor support were listed in the higher category of average time spent on the website compared with the lower percentage of studies evaluating interventions that did not include peer or counselor support, and that the difference in number of interventions listed in the higher category was at least 35%. Both email/phone contact with visitors and updates of the intervention website were related to more average log-ins on the intervention websites, indicated by the higher number of studies on interventions that included these elements listed in the higher average log-in categories, as compared with interventions without these elements.

**Table 3:** Listing of studies by potential exposure-promoting elements (interactive behavior change strategies, interactive elements, peer support, and counselor support) and the result of exposure measures (also see Table 4 below)

Exposure Measures		Interactive Behavior Change Strategies				Interactive Elements				Peer Support				Counselor Support			
		0-3 Strategies		≥ 3 Strategies		Yes		No		Yes		No		Yes		No	
		n	Study Number^a^	n	Study Number^a^	n	Study Number^a^	n	Study Number^a^	n	Study Number^a^	n	Study Number^a^	n	Study Number^a^	n	Study Number^a^
**Percent of participants completing modules/intervention during first visit (n = 16)**																	
	Total n for element and exposure	15		1		10		6		4		12		3		13	
	< 70%	5	35a, 35b, 39, 52, 59			4	35a, 35b 52, 59	1	39	2	35a, 52	3	35b, 39, 59	2	35a, 39	3	35b, 52, 59
	70%-90%	6	14a, 29, 50, 51a, 51b, 53			3	14a, 51a, 53	3	29, 50, 51b	1	53	5	14a, 29, 50, 51a, 51b	1	29	5	14a, 50, 51a, 51b, 53
	> 90%	4	10, 14b, 43, 64a	1	60	3	10, 43, 64a	2	14b, 60	1	10	4	14b, 43, 60, 64a			5	10, 14b, 43, 60, 64a
**Average duration of visits in minutes (n = 16)**																	
	Total n for element and exposure	8		8		11		5		9		7		6		10	
	< 10 minutes	5	7b, 13, 40a, 42b, 56	5	5, 7a, 8, 40b, 62	7	5, 7a, 7b, 8, 13, 42b, 56	3	40a, 40b, 62	4	5, 40a, 40b, 62	6	7a, 7b, 8, 13, 42b, 56	2	13, 40a	8	5, 7a, 7b, 8, 40b, 42b, 56, 62
	10-20 minutes	3	42a, 44a 44b	3	37, 54a, 54b	4	37, 42a, 54a, 54b	2	44a, 44b	5	37, 42a 44a, 54a 54b	1	44b	4	37, 42a, 44a, 44b	2	54a, 54b
**Average number of pages visited (n = 4)**																	
	Total n for element and exposure	2		2		4		0		1		3				3	
	< 10 pages	1	49			1	49					1	49			1	49
	10-50 pages	1	15	1	8	2	8,15					2	8,15	1	37	2	8,15
	> 50 pages			1	37	1	37			1	37						
**Average number of log-ins on website (n = 27)**																	
	Total n for element and exposure	16		11		19		8		15		12		11		16	
	1-5 times	6	3b, 13, 15, 40a, 42a, 42b	3	40b, 54a, 54b	6	13, 15, 42a, 42b, 54a, 54b	3	3b, 40a, 40b	5	40a, 40b, 42a, 54a, 54b	4	3b, 13, 15, 42b	3	13, 40a, 42a	6	3b, 15, 40b, 42b, 54a, 54b
	5-10 times	3	3a, 26b, 36	1	6b	3	3a, 26b, 36	1	6b	2	3a, 36	2	6b, 26b	2	3a, 36	2	6b, 26b
	> 10 times	7	16, 21, 26a, 28a, 28b, 32, 33	7	5, 6a, 12a, 12b, 22, 37, 62	10	5, 6a, 12a, 12b, 16, 21, 26a, 28a, 28b, 37	4	22, 32, 33, 62	8	5, 6a, 16, 26a, 28a, 28b, 37, 62	6	12a, 12b, 21, 22, 32, 33	6	12a, 12b, 21, 26a, 28a, 37	8	5, 6a, 16, 22, 28b, 32, 33, 62

**Percent of participants who revisited website (n = 8)**																	
	Total n for element and exposure	3		5		5		3		5		3		3		5	
	< 20%	2	48, 61	1	9	1	61	2	9, 48	1	48	2	9,61			3	9,48,61
	20%-50%	1	31	2	11a, 41	2	11a, 41	1	31	2	11a, 41	1	31	1	41	2	11a,31
	> 50%			2	34, 37	2	34, 37			2	34, 37			2	34,37		
**Percent of participants who completed all modules in multiple visits (n = 10)**																	
	Total n for element and exposure	4		6		6		4		5		5		1	9	9	
	< 20%	2	52, 53			2	52, 53			2	52, 53					2	52, 53
	20%-50%			4	6b, 62, 63a, 63b	2	63a, 63b	2	6b, 62	1	62	3	6b, 63a, 63b			4	6b, 62, 63a, 63b
	> 50%	2	32, 33	2	6a, 37	2	6a, 37	2	32, 33	2	6a, 37	2	32, 33	1	37	3	6a, 32, 33

^a^ The numbering of studies corresponds with the numbering of studies in Table 2 and the Multimedia Appendices: physical activity study numbers are 1-12; nutrition, 13-16; weight management, 17-30; smoking cessation, 31-48; alcohol consumption, 49-57; and multi-behaviors, 58-64. The letters a and b are used when in a study different Internet interventions are described (see Multimedia Appendix 1 and Multimedia Appendix 2.)

**Table 4:** Listing of studies by potential exposure-promoting elements (email/phone contact, updates, and intervention incentives) and the result of exposure measures^a^

Exposure Measures		Email/Phone Contact				Update				Intervention Incentive			
		Yes		No		Yes		No		Yes		No	
		n	Study Number^a^	n	Study Number^a^	n	Study Number^a^	n	Study Number^a^	n	Study Number^a^	n	Study Number^a^
**Percent of participants completing modules/intervention during first visit (n = 16)**													
	Total n for element and exposure	6		10		4		12		0		16	
	< 70%	3	35a, 39, 52	2	35b, 59	2	35a, 52	3	35b, 39, 59			5	35a, 35b, 39, 52, 59
	70%-90%	2	29, 53	4	14a, 50, 51a, 51b	2	29, 53	4	14a, 50, 51a, 51b			5	14a, 50, 51a, 51b, 53
	> 90%	1	64a	4	10, 14b, 43, 60			5	10, 14b, 43, 60, 64a			6	10, 14b, 29, 43, 60, 64a
**Average duration of visits in minutes (n = 16)**													
	Total n for element and exposure	10		6		4		12		2		14	
	< 10 minutes	6	5, 7a, 7b, 8, 13, 62	4	40a, 40b, 42b, 56	3	7a, 8, 13	7	5, 7b, 40a, 40b, 42b, 56, 62	1	13	9	5, 7a, 7b, 8, 40a, 40b, 42b, 56, 62
	10-20 minutes	4	37, 42a, 44a, 44b	2	54a, 54b	1	42a	5	37, 44a, 44b, 54a, 54b	1	37	5	42a, 44a, 44b, 54a, 54b
**Average number of pages visited (n = 4)**													
	Total n for element and exposure	3		1		1		3		2		2	
	< 10 pages			1	49	1	8	1	49			1	49
	10-50 pages	2	8, 15					1	15	1	15	1	8
	> 50 pages	1	37					1	37	1	37		
**Average number of log-ins on website (n = 27)**													
	Total n for element and exposure	18		9		11		16		5		22	
	1-5 times	4	3b, 13, 15, 42a	5	40a, 40b, 42b, 54a, 54b	2	13, 42a	7	3b, 15, 40a, 40b, 42b, 54a, 54b	2	13, 15	7	3b, 40a, 40b, 42a, 42b, 54a, 54b
	5-10 times	2	3a, 36	2	6b, 26b	1	3a	3	6b, 26b, 36			4	3a, 6b, 26b, 36
	> 10 times	12	5, 6a, 12a, 12b, 16, 21, 22, 26a, 32, 33, 37, 62	2	28a, 28b	8	12a, 12b, 16, 21, 28a, 28b, 32, 33	6	5, 6a, 22, 26a, 37, 62	3	12a, 16, 37	11	5, 6a, 12b, 21, 22, 26a, 28a, 28b, 32, 33, 62

**Percent of participants who revisited website (n = 8)**													
	Total n for element and exposure	7		1		1		7		1		7	
	< 20%	2	48, 61	1	9	1	61	2	9, 48			3	9, 48, 61
	20%-50%	3	11a, 31, 41					3	11a, 31, 41			3	11a, 31, 41
	> 50%	2	34, 37					2	34, 37	1	37	1	34
**Percent of participants who completed all modules in multiple visits (n = 10)**													
	Total n for element and exposure	7		3		6		4		1		9	
	< 20%	2	52, 53			2	52, 53					2	52, 53
	20%-50%	1	62	3	6b, 63a, 63b	2	63a, 63b	2	6b, 62			4	6b, 62, 63a, 63b
	> 50%	4	6a, 32, 33, 37			2	32, 33	2	6a, 37	1	37	3	6a, 32, 33

^a^ The numbering of studies is explained in the footnote to Table 3.

## Discussion

Nonoptimal exposure to Internet interventions has been pointed out as a major concern in the field of development, evaluation, and implementation of Internet interventions [19]. According to the diffusion of innovations theory [25], characteristics of (potential) users and characteristics of an intervention (ie, the innovation) are associated with adoption and implementation of interventions. The present review is one of the first to systematically investigate which specific characteristics of an Internet intervention can be associated with better exposure to the intervention and its contents. The study was qualitative in nature and allowed us to point out indications of possible patterns in associations between intervention characteristics and exposure. Of the categories of potential exposure-improving intervention elements that we distinguished in the review (the number of interactive behavior change strategies used, and whether the intervention included interactive elements, peer support, counselor support, email and/or phone contact, update of the intervention website, and intervention incentives), peer and counselor support were related to a longer visit duration, and email/phone contact and update of the intervention website were related to a higher frequency of website log-ins. There were a large variety of potentially exposure-increasing elements applied in the various interventions, and there was a large variety and little consistency in the exposure measures that were reported.

In previous studies, interactively delivered educational content, such as the provision of computer-tailored feedback and goal setting, has been indicated as a potentially exposure-improving element [26-28,31]. The active involvement required for using interactive elements, the personal relevance of feedback, and goals generated may result in more involvement in and better exposure to an intervention program. In this study, however, we did not find an association between the number of interactive behavior change strategies and exposure. This may be due to the fact that there was little variability in the use of these elements. For example, in about three quarters of the interventions, some type of tailored feedback was provided. What this review showed is that there was a marked difference in the use of other interactive educational content between the interventions for the various target behaviors. This may reflect differences in the importance of the underlying determinants and change methods needed to facilitate effective and maintained change in the various behaviors. It may also reflect that Internet applications are more advanced for the promotion of some of the health related behaviors (eg, promotion of physical activity, weight management, and smoking cessation) than for others.

Peer support was offered more often in weight management, alcohol, and smoking cessation interventions as compared with the other behaviors. Based on our criteria, peer support was related to more time spent on the intervention website. This does not necessarily mean, however, that visitors are exposed to and actively engaged in the intervention content, but they may at least be chatting about their target behavior, for example, in a forum or a chat room. Furthermore, it should be noted that previous studies reported that peer support is used to a limited extent and that not all visitors may use peer support [26,27,31]. Peer support was, for example, more often sought by smoking quitters than by visitors that continued smoking [70,72], and women have been found to be more likely to post more messages than men on a message board about smoking cessation [71].

Counselor support was more often a distinct part of the weight management and smoking cessation interventions. The results indicate that counselor support was related to a longer website visit. Although there were an insufficient number of interventions in our study to draw any conclusions about the potential relation between counselor support and revisiting intervention websites, there may be a positive relation. These findings may add positively to the results of previous single studies where inconsistent findings were reported for the relation of counselor support and submission of dietary reports. Tate et al [63], for example, showed that additional human email counseling resulted in higher online diary submissions, whereas Webber et al [64] found the opposite.

Nearly half of the interventions sent email/phone prompts to encourage revisits. Next to that, weight management interventions made more use of emails sent by counselors, whereas physical activity and smoking cessation interventions used automatically generated emails to send intervention content. This review shows that email/phone contact might indeed be useful in promoting repeated visits as has already been indicated in single studies addressing this topic. Furthermore, the postulation that regular updates of the intervention website would be related to repeated visits seems to be supported by the findings of this review. There is growing evidence that repeated website visits are necessary to achieve sustainable changes [22-24]. However, disappointing results regarding revisiting have been published, as website visits tend to decrease sharply after the initial weeks of participation [4,23,39]. It is, therefore, promising that email prompts and regular updates of intervention content may contribute to more visits, since these are relatively easy to implement exposure-promoting strategies.

Another important finding in this review is that there was a large variety in the report of objective exposure measures but also that many studies that did not report exposure data at all. We had to exclude 29 publications solely because they did not present any objective exposure measures. The number of log-ins on the intervention website was the most frequently reported exposure measure, but this measure was presented in different ways, which limited the options of pooling the data. Other often-presented exposure measures were completion of the initial visit, visit duration, and completion of the intervention program in case revisits were required. It is not only important that objective exposure measures (eg, starting intervention, completing modules/intervention, frequency of visiting, and duration of visit) are presented in studies evaluating Internet interventions [32,101], but it is also important that these measures are presented in a standardized way. Furthermore, for the purpose of systematic reviews, it is very important that accurate and complete descriptions of intervention content and interactive applications are provided in the future. This would make it possible to compare and pool different studies and enlarge the strength of the conclusions that can be drawn. In addition, objective exposure measures should be linked to visitor characteristics to get a more thorough impression about who is reached with what kind of intervention and to what extent. Furthermore, this registration on the individual level would also make it possible to study possible mediating effects of exposure to these objective exposure outcome measures.

To be able to relate the potentially exposure-improving intervention characteristics with exposure measures, we developed a matrix containing both elements. We listed all studies in this matrix by categorizing them according to, for example, the number of interactive behavior change strategies used and the presence of peer or counselor support and the result of the exposure outcome. From this qualitative integrative approach, we derived that peer support was associated with a longer stay on the website, whereas email/phone contact and update of the intervention website were related to more log-ins on the intervention website. We did not find an indication of better exposure to the intervention for the other categories of potential exposure-enhancing intervention characteristics, even if these have been indicated as such in previous studies [26-31]. This is also in contrast with the findings of individual studies in which a more extensive version of an intervention with more interactive characteristics was compared with a more basic version. A more interactive intervention resulted, for example, in a longer visit to the intervention [31,77] and in more log-ins on the intervention website [28,31,62]. One possible reason for not finding differences in exposure according to the use of more as compared with fewer interactive behavior change strategies is the way in which we divided the interventions (< 3 or ≥ 3 interactive behavior change strategies) and that we pooled all the interventions targeting different health-related behaviors together.

The findings of our study are partly in line with the only other study that investigated the same topic among adolescents and young adults [102]. Similar to our study, they also found a heterogeneity of exposure measures and identified different exposure-increasing methods and strategies, such as tailored feedback, use of interactive elements, email support, and reminders. Furthermore, single studies showed that more interactive interventions resulted in a higher exposure to the intervention content than a basic version. Nevertheless, we have to keep in mind that younger people use the Internet differently than adults [3,103].

### Limitations

There are some limitations to this review study that need to be mentioned. The search strategies were limited to include only peer-reviewed English language publications. Therefore, we could have missed important “gray literature” and publication in languages other than English. Next, for this review we relied on the information that was provided in the published literature regarding the description of the intervention and identification of potentially exposure-promoting methods and strategies. Some of the intervention descriptions were very brief, and even the more extensive descriptions available in the literature may not always have been complete. Therefore, we may have missed some of the potential exposure-promoting elements that an intervention contained. In addition, this review can be qualified as a qualitative review as the extracted data from the included studies were summarized and not statistically pooled, which limits the strength of the conclusions that can be drawn. Finally, the used cutoff points for making a ranking within the categories of potential exposure-promoting interventions elements (ie, < 3 or ≥ 3 interactive behavior change strategies, and yes vs no interactive elements) may not have been sensitive enough to detect differences in exposure.

### Conclusion

The studies included in this review showed that in the Internet interventions currently available, a wide variety of potentially exposure-improving methods and strategies were used. These methods and strategies were markedly different for the healthy lifestyle behaviors that were studied. Also, a large variety of objective exposure outcome measures were used and there was a lack of consistency in exposure measures reported. Peer support, counselor support, email/phone contact with visitors through sending intervention content and prompts and updates of the intervention website were indicated to result in a longer visit and more log-ins on the website, respectively. More research is needed to gain insight into how intervention characteristics can be used to improve exposure to Internet interventions. More accurate and consistent description of intervention content and more consistency in the report of objective exposure outcomes are recommended. This will enable researchers to better assess associations between intervention characteristics and exposure to health behavior change Internet interventions in the future.

## Multimedia Appendix 1

Overview of study and intervention characteristics and objective outcome measures of exposure to Internet interventions by behavior

## Multimedia Appendix 2

Summary of studies included in this review: target behavior, study characteristics, brief description of Internet intervention content and duration, theory, number of study participants and characteristics of study population

## Abbreviations

RCT: randomized controlled trial
