# Supplementary material for: Which Intervention Characteristics are Related to More Exposure to Internet-Delivered Healthy Lifestyle Promotion Interventions? A Systematic Review
Source: J Med Internet Res. 2011 Jan 6;13(1):e2. doi: 10.2196/jmir.1639 (PMC3221341; doi:10.2196/jmir.1639)
Supplement: Supplementary file 1 [file jmir_v13i1e2_app1.pdf]

Multimedia Appendix 1 Overview of study and Internet characteristics and objective outcome measures of exposure to Internet interventions presented to behavior

Study[a], country	Target behavior, target group (N)	Potential exposure promoting elements, within main categories[b]	Objective outcome measure regarding exposure to Internet intervention[b]
**A. Physical activity**			
1. Carr (2008) [36], USA	**Target behavior:** physical activity<br>**Target group:** sedentary overweight non-smoking adults (N=32) with a BMI between 18-40	**IBC:** 1. goal setting; 2. activity planning; 3. self-monitoring; 4. feedback on progress<br>**IE:** virtual partner stories, quizzes, website links<br>**PS:** -<br>**CS:** weekly/bi-weekly e-mail/phone contact with facilitator<br>**EP:** -<br>**UD:** provision of new lessons<br>**II:** -	**Login:** every 11th day login during the intervention<br>**IBC:** avg. 13 of 44 online journal activities (30%)
2. Dunton (2008) [37], USA	**Target behavior:** physical activity<br>**Target group:** healthy and racially/ ethnically-diverse females (N=156) aged 21-65	**IBC:** 1. feedback on performance, cognitive constructs, and barrier identification and solution<br>**IE, PS, CS:** -<br>**EP:** 10 weekly follow-up e-mail newsletters with generic PA promotion information<br>**UD, II:** -	**Login:** 0 times 5%; 1-2 times: 21%; 3-5 times: 37%; 6-10 times: 29%; >10 times: 8%<br>**EP:** avg. 7.44 out of 10 ± 4.1 e-mails opened; 6.65 out of 25 ± 6.33 website links embedded in e-mails were opened (27%)
3. Ferney (2008) [28], Australia	**Target behavior:** walking and overall physical activity<br>**Target group:** adults (N=106) aged 45-60 who did not meet current PA guidelines	**a. IBC:** 1. goal setting; 2. self-monitoring<br>**IE:** target heart rate calculator, searchable database of local PA opportunities, website links<br>**PS:** bulletin board<br>**CS:** possibility to e-mail counselor<br>**EP:** weekly/bi-weekly/maintenance e-mails with tailored feedback<br>**UD:** bi-weekly updated news items<br>**II:** -	[d]**Login:** avg. 8.2 logins ± 9.0<br>**IBC:** 13% used self-monitoring tool**;** 52% completed at least 1 tailored quiz, avg. 2.2 ± 1.4 quizzes<br>**PS:** 1 participant posted message on bulletin board<br>**CS:** 25% e-mailed counselor
		**b. IBC, IE, PS, CS:** -<br>**EP:** e-mail with non-tailored advice<br>**UD, II:** -	[d]**Login:** avg. 2.8 logins ± 2.4
4. Herman (2006) [38], USA	**Target behavior:** physical activity<br>**Target group:** employees of multinational information technology company (N=67,324)	**IBC:** 1. goal setting; 2. self-monitoring; 3. feedback on progress<br>**IE, PS:** -<br>**CS:** ask the expert<br>**EP:** e-mails with educational and motivational content<br>**UD:** -<br>**II:** mementos and cash rebate	**Access program content:** 53%

5. Hurling (2007) [39], UK	**Target behavior:** total and moderate to vigorous physical activity<br>**Target group:** adults (N=77) aged 30-55 with a BMI of 19-30 who were not vigorously active	**IBC:** 1. feedback on performance, cognitive constructs, and barrier identification and solutions; 2. activity planning; 3. self-monitoring; 4. feedback on progress<br>**IE:** information library<br>**PS:** message board<br>**CS:** -<br>**EP:** e-mail and/or mobile phone prompts (optional), e-mail and/or mobile phone messages with motivational content (optional)<br>**UD, II:** -	[d]**Visit duration:** avg. 7.5 min ± 0.9<br>[d] **Login**: avg. 2.9 logins ± 0.5 per week resulting in avg. 26.1 logins for 9 weeks<br>**IBC:** use of activity charts (showing the accelerometer feedback data), schedule (weekly exercise planner) by at least 33%<br>**PS:** use of chat-room style message board by at least 33%
6. Hurling (2006) [40], UK	**Target behavior:** physical activity/exercise<br>**Target group:** adult employees (N=66) aged 23-54 years	**a. IBC:** 1. feedback on performance, cognitive constructs, and barrier identification and solutions; 2. activity planning; 3. self-monitoring; 4. feedback on progress<br>**IE:** information library<br>**PS:** message board<br>**CS:** -<br>**EP:** e-mail and/or mobile phone prompts (optional), e-mail and/or mobile phone messages with motivational content (optional)<br>**UD, II:** -	[d]**Login:** avg. 1.4 logins per week resulting in avg. 14 logins for 10 weeks<br>[d]**Completion whole intervention:** 75%
		**b. IBC:** 1. feedback on barrier identification and solution; 2. self-monitoring; 3. feedback on progress<br>**IE, PS, CS, EP, UD, II:** -	[d]**Login:** avg. 1.0 logins per week; avg. 10 logins for 10 weeks<br>[d]**Completion whole intervention:** 43%
7. Lewis (2008) [41], Marcus (2007) [42], USA	**Target behavior:** physical activity and exercise<br>**Target group:** healthy sedentary adults (N=249) aged 18 and older who were ≤ 90 minutes per week physical active	**IBC:** 1. feedback on performance and cognitive constructs; 2. goal setting; 3. self-monitoring; 4. feedback on progress<br>**IE:** website links<br>**PS, CS:** -<br>**EP:** e-mail as prompts<br>**UD:** monthly tailored feedback reports, daily update with tip of the day<br>**II:** -	[d]**Duration visit:** avg. 7.1 min per session; total avg. 356 min in one year<br>**Login:** median 50 logins
		**b. IBC:** 1. self-monitoring<br>**IE:** website links<br>**PS, CS:** -<br>**EP:** e-mail as prompts<br>**UD, II:** -	[d]**Duration visit:** avg. 6.8 min per session; total avg. 260 min in one year<br>**Login:** median 38 logins
8. Leslie (2005) [16], Australia	**Target behavior:** physical activity and exercise<br>**Target group:** academic and general staff of medium-sized university (N=655)	**IBC:** 1. feedback on cognitive constructs; 2. goal setting; 3. activity planning<br>**IE:** target heart rate calculator, rotating photo images of PA options<br>**PS, CS:** -<br>**EP:** 4 bi-weekly personalized stage-targeted e-mails<br>**UD:** daily update with tip of the day<br>**II:** -	**Landing website:** 4114 hits<br>**Access program content:** 46%<br>[d]**Duration visit:** avg. 9 min<br>[d]**Pages visited:** avg. 18 pages<br>**IBC:** 66% completed at least on stage-based quiz
9. Plotnikoff (2006) [43], Canada	**Target behavior:** raise awareness for physical activity<br>**Target group:** general national population (N=3175)	**IBC:** 1. goal setting; 2. activity planning; 3. self-monitoring; 4. feedback on progress<br>**IE, PS, CS, EP, UD, II:** -	[d]**Revisit website:** 15%

10. Spittaels (2006) [44], Belgium	**Target behavior:** physical activity<br>**Target group:** visitors (N=55) of a university hospital aged 20 to 55	**IBC:** 1. feedback on performance, cognitive constructs, and barrier identification; 2. activity planning<br>**IE:** website links<br>**PS:** forum<br>**CS, EP, UD, II:** -	**Access program content:** 28% (46% distributed with personal contact, 7% without personal contact)<br>[d]**Completion intervention first visit:** 90% (89% distributed with personal contact, 100% without personal contact)<br>**IBC/IE:** 22% of the visitors used one or more supplementary parts
11. Spittaels (2007) [45], Belgium	**Target behavior:** physical activity<br>**Target group:** adults aged 20 to 55 (N=434)	**a. IBC:** 1. feedback on performance and cognitive constructs; 2. activity planning; 3. feedback on progress<br>**IE:** website links<br>**PS:** forum<br>**CS:** -<br>**EP:** 7 non-tailored e-mails as prompts to revisit<br>**UD:** new tailored advice<br>**II:** -	[d]**Revisit website:** 31% to receive second tailored advice
		**b. IBC:** 1. feedback on performance and cognitive constructs; 2. activity planning<br>**IE:** website links<br>**PS:** forum<br>**CS, EP, UD, II:** -	
12. Steele (2007) [46, 47], Australia	**Target behavior:** moderate intensity and lifestyle physical activity<br>**Target group:** inactive adults (N=192) aged 18 and older being functionally mobile	**a. IBC:** 1. feedback on cognitive constructs; 2. goal setting; 3. activity planning; 4. self-monitoring<br>**IE:** quizzes, multimedia video clips<br>**PS:** -<br>**CS:** access to online support person<br>**EP:** weekly e-mail as prompts<br>**UD:** weekly modules<br>**II:** opportunity to receive incentives	[d]**Login:** avg. 11.8 logins [range 2-90]
		**b. IBC:** 1. feedback on cognitive constructs; 2. goal setting; 3. activity planning; 4. self-monitoring<br>**IE:** multimedia video clips<br>**PS:** -<br>**CS:** access to online support person<br>**EP:** weekly e-mail as prompts<br>**UD:** weekly modules<br>**II:** -	[d]**Login:** avg. 11.5 logins [range 2-102]
**B. Nutrition**			
13. Buller (2008) [48], Woodall 2007 [49], USA	**Target behavior:** fruit and vegetable consumption<br>**Target group:** adults (N=755) living in specific area for at least 6 months and being older than 18 years	**IBC:** -<br>**IE:** website links, community directory<br>**PS:** -<br>**CS:** community outreach trainer for questions/problems website<br>**EP:** email as prompts<br>**UD:** new or updated information<br>**II:** every 2 months small gift	**Access intervention content:** 51%<br>[d]**Duration visit:** avg. of 22.2 minutes total time [range 0-322.7] meaning avg. of 6.7 min per login<br>[d]**Login:** avg. of 3.3 logins [range 1-39]<br>**E-mail**: 23% responded to at least one of the e-mails by logging in within 5 days of e-mail was sent; 6 participants responded to all of the messages; those who responded to at least one email, 51% responded to half or more of the messages, while 49% responded to fewer than half.
14. Huang (2006) [50], Australia	**Target behavior:** purchases with saturated fat<br>**Target group:** adult consumers (N=497) using a commercial online Internet supermarket shopping site	**IBC:** feedback on performance<br>**IE:** point of purchase decision making<br>**PS, CS, EP, UD, II:** -	**Landing website:** total of 11% for both interventions<br>[d]**Completing first visit:** 89%<br>**Login:** median of 3 shopping episodes [range 1-20]
		**IBC, IE, PS, CS, EP, UD, II:** -	[d]**Completing first visit:** 94%

15. McNeill (2007) [51], USA	**Target behavior:** fruit and vegetable consumption<br>**Target group:** adults (N=52) residing in low-income multiethnic neighborhoods	**IBC:** -<br>**IE:** recipes database<br>**PS, CS:** -<br>**EP:** e-mail as prompt, and e-mail with feedback on performance and tips on increasing consumption<br>**UD:** -<br>**II:** raffle for a small incentive	**Access website content:** 75%<br>[d]**Pages visited:** avg. 24.5 pages out of 192 distinct pages<br>[d]**Login:** avg. 3.8 logins<br>**E-mail:** 56% login after first reminder; 27% after second reminder; 56% after final reminder
16. Papadaki (2005) [52], Papadaki (2006) [53], Scotland	**Target behavior:** Consumption of four key components of the Mediterranean diet<br>**Target group:** healthy females university employees (N=72) aged 25 to 55	**IBC:** -<br>**IE:** self-assessment quizzes, recipe section<br>**PS:** bulletin board<br>**CS:** -<br>**EP:** 6 e-mails with feedback letters on performance, cognitive constructs, barrier identification and solution, progress, and progress; weekly e-mails as prompts including tips for relevant sections at the website with respect to goal<br>**UD:** regular updates with tip of the day and new recipes<br>**II:** -	**Landing website:** avg. 150 hits each month<br>[d]**Login**: avg. 15.5 logins
**C. Weight management**			
17. Cussler (2008) [54], USA	**Target behavior:** weight maintenance through diet, physical activity and weight gain prevention<br>**Target group:** premenopausal women (N=135) aged 40 to 55 with BMI between 25 and 38, non smokers	**IBC:** 1. self-monitoring; 2. feedback on progress<br>**IE:** communication tools, website links<br>**PS:** self-organized support groups meeting online, bulletin board, chat rooms<br>**CS:** optional counselor support through e-mail, bulletin board and chat<br>**EP:** e-mail as intervention content<br>**UD:** new added information/articles<br>**II:** several web-based incentive programs	**IBC:** diet log: 90%, avg. 53.4 ± 62.3 [range 0-299]; weekly weight log: 100%, avg. 26.9 ± 19.9 [range 1-69]; physical activity log: 84%, avg. 67.5 ± 76.3 [range 0-294]; 'your week' log: 71%, avg. 9.2 ± 12.2 [range 0-42]<br>**PS:** 81%; avg. 84.3 ± 157.1 [range 0-835]
18. Glasgow (2007) [21], USA	**Target behavior:** weight loss through nutrition and physical activity<br>**Target group:** health plan members (N=2311) with BMI < 30 for general membership and < 25 for those with chronic disease	**a. IBC:** 1. feedback on performance, cognitive constructs, and barrier identification and solution; 2. goal setting; 3. action planning<br>**IE, PS, CS:** -<br>**EP:** 6 e-mail as prompt to view follow-up action plans and tailored newsletters<br>**UD:** 6 tailored newsletters<br>**II:** -	**Access program content:** 13% accessed initial program content; 6% accessed extended program content
		**b. IBC:** 1. feedback on performance, cognitive constructs, barrier identification and solution; 2. goal setting; 3. action planning<br>**IE, PS, CS:** -<br>**EP:** 3 e-mails as prompt to view follow-up action plans<br>**UD:** 3 tailored newsletters<br>**II:** -	**Access program content:** 62% accessed initial program content; 25% accessed extended program content
		**c. IBC:** 1. feedback on performance, cognitive constructs, barrier identification and solution; 2. action planning<br>**IE, PS, CS:** -<br>**EP:** 6 e-mails as prompt to view follow-up action plans and tailored newsletters<br>**UD:** 6 tailored newsletters<br>**II:** -	**Access program content:** 19% accessed initial program content; 8% accessed extended program content

		**d. IBC:** 1. feedback on performance, cognitive constructs, barrier identification and solution; 2. action planning<br>**IE, PS, CS:** -<br>**EP:** 3 e-mails as prompt to view follow-up action plans<br>**UD:** 3 tailored newsletters<br>**II:** -	**Access program content:** 90% accessed initial program content; 49% accessed extended program content
19. Gold (2007) [55], USA[c]	**Target behavior:** weight loss through reducing calorie intake and increase aerobic activity<br>**Target group:** adults (N=124) aged 18 and older with a BMI between 25 and 39.9	**a. IBC:** 1. feedback on performance; 2. goal setting; 3. self-monitoring; 4. feedback on progress<br>**IE:** contests, menu/recipe planner, food and exercise-calorie database, target heart rate, BMI calculator<br>**PS:** bulletin boards, e-mail possibilities with peers<br>**CS:** weekly/bi-weekly therapist-led chat meetings; weekly/bi-weekly e-mails from therapist with feedback on completed assignments<br>**EP:** weekly/biweekly e-mails with intervention content from counselor<br>**UD:** weekly new lesson; weekly updated story, new flashes and tips; periodically updated motivation page and local events guide<br>**II:** periodically contests with prizes	**Login:** median 193/0-6 [range 120-309]; 90/6-12 months [range 21-154]<br>**IBC:** self-reported weight median 24/0-6 months [range 20-25]; 8/6-12 months [range 2-12]<br>**CS**: attendance online meeting median 21/0-6 months [range 19-23); 11/6-12 months [range 6-14]
		**b. IBC:** 1. feedback on cognitive constructs; 2. self-monitoring; 3. feedback on progress<br>**IE:** FAQ with expert responses<br>**PS:** chat-rooms, discussion boards, mentor section to team up with more experienced member<br>**CS:** professional facilitated online meetings<br>**EP, UD, II:** -	**Login:** median 47/0-6 months [range 25-65]; 14/6-12 months [range 8-23]<br>**IBC:** self-reported weight median 16/0-6 months [range 8-22]; 8/6-12 months [range 2-13]<br>**CS:** attendance online meeting median 1/0-6 months [range 0-3]; 0/6-12 months [range 0-0]
20. Harvey-Berino (2002) [56], USA	**Target behavior:** weight loss maintenance through modification of eating and exercise habits<br>**Target group:** overweight adults (N=122) aged 18 and older with a BMI >25	**IBC:** 1. self-monitoring<br>**IE:** short videos from group therapist<br>**PS:** self-initiated chat room meetings, bulletin board<br>**CS:** counselor support through bi-weekly chat sessions and e-mails<br>**EP:** bi-weekly e-mails from group therapist<br>**UD:** -<br>**II:** participation in weekly $25-lottery and opportunity to earn points for lottery tickets	**IBC:** submitting online date 19%<br>**CS:** attendance chat sessions 39%
21. Hunter (2008) [57], USA	**Target behavior:** weight gain prevention and weight loss through restricting calorie intake and fat intake and increasing physical activity<br>**Target group:** air force personnel (N=446) aged 18 to 65 with a BMI > 25 for women and > 27.5 for men and remain in local area for 1 year	**IBC:** 1. goal setting; 2. self-monitoring<br>**IE:** quizzes<br>**PS:** -<br>**CS:** internet counselor provided weekly feedback on performance and progress<br>**EP:** internet counselor made two brief motivational interviewing telephone calls<br>**UD:** weekly lessons<br>**II:** -	[d]**Login:** avg. 49.1 logins [range 1-707]
22. McConnon (2007) [58], UK	**Target behavior:** Weight loss through dietary and physical activity<br>**Target group:** adults (N=221) aged 18 to 65 with a BMI > 30	**IBC:** 1. feedback on performance; 2. self-monitoring; 3. feedback on progress<br>**IE, PS, CS:** -<br>**EP:** e-mails as prompts<br>**UD, II:** -	[d]**Login:** avg. 15.8 times ± 15.2 [range 1-77]

23. McCoy (2005) [59], Australia	**Target behavior:** weight loss through changing physical activity and dietary behavior<br>**Target group:** adults (N=808) of the general population	**IBC:** 1. feedback on cognitive constructs; 2. action planning<br>**IE, PS, CS, EP, UD, II:** -	**Access program content:** 68%<br>**Pages visited:** participants viewed in total 83,111 pages<br>**Login:** participants used the program in total 6058 times with avg. 29 homepage visits per participant<br>**IBC:** participants used in total the wellness record page 1792 times, the exercise planner 2487 times, and the diet planner 1344 times
24. Micco (2007) [60], USA[c]	**Target behavior:** weight loss through changing eating and exercise behavior<br>**Target group:** Adults (N=123) aged 18 and older with a BMI between 25 and 39.9, non-smoking	**a. IBC:** 1. feedback on performance; 2. goal setting; 3. self-monitoring; 4. feedback on progress<br>**IE:** contests, menu/recipe planner, food and exercise-calorie database, target heart rate, BMI calculator<br>**PS:** bulletin boards, e-mail possibilities with peers<br>**CS:** weekly/bi-weekly therapist-led chat meetings; weekly/bi-weekly e-mails from therapist with feedback on completed assignments<br>**EP:** weekly/biweekly e-mails with intervention content from counselor<br>**UD:** weekly new lesson; weekly updated story, new flashes and tips; periodically updated motivation page and local events guide<br>**II:** periodically contests with prizes	**Login:** 0-6 months total of 223 hits; 7-12 months total of 99 hits<br>**IE:** use BMI calculator avg. 2.6 times ± 3.0<br>**CS:** attendance online meetings 0-6 months 76% ± 21%; 7-12 months: 58% ± 33%
		**b. IBC:** 1. feedback on performance; 2. goal setting; 3. self-monitoring; 4. feedback on progress<br>**IE:** contests, menu/recipe planner, food and exercise-calorie database, target heart rate, BMI calculator<br>**PS:** bulletin boards, e-mail possibilities with peers<br>**CS, EP:** -<br>**UD:** weekly new lesson; weekly updated story, new flashes and tips; periodically updated motivation page and local events guide<br>**II:** periodically contests with prizes	**Login:** 0-6 months total of 206 hits; 7-12 months total of 90 hits<br>**IE:** use BMI calculator avg. 1.0 times ± 1.5<br>**CS:** attendance online meetings 0-6 months 54% ± 14%; 7-12 months: 55% ± 34%
25. Petersen (2008) [61], USA	**Target behavior:** weight management by creating life long habits<br>**Target group:** Employees of a multinational information technology company (N=7743)	**IBC:** 1. feedback on performance; 2. goal setting; 3. self-monitoring; 4. feedback on progress<br>**IE:** interactive tools, e.g. meal planners, grocery lists, serving size calculator, information library<br>**PS:** message boards<br>**CS:** expert assistance<br>**EP:** weekly e-mail newsletters, e-mails individualized to visitors' goals<br>**UD:** -<br>**II:** 'fictive' points that can be earned in interaction with website features	**Access program content:** 6%<br>**Login:** 0-2 days 42%; 3-11 days 36%; ≥12 days 22%
26. Tate (2001) [62], USA	**Target behavior:** weight loss through calorie restriction and increased physical activity<br>**Target group:** Overweight hospital employees (N=91) aged 18 to 60 with a BMI of 25 to 36	**a. IBC:** 1. self-monitoring<br>**IE:** website links<br>**PS:** bulletin board<br>**CS:** weekly e-mail from therapist with feedback on progress, recommendations and strategies for improvement, answers on questions and encouragement<br>**EP:** weekly behavioral weight loss lesson, and personal e-mail to motivate to continue for participants not sending log<br>**UD, II:** -	**IBC:** mean submission of 13.65 ± 6.4 self-monitoring diaries<br>**PS:** 28% posted a note on bulletin board [range 1-7]<br>[d]**Login:** avg. 19 logins ± 10.9
		**b. IBC:** 1. self-monitoring<br>**IE:** website links<br>**PS, CS, EP, UD, II:** -	[d]**Login:** avg. 8.5 logins ± 10.4

27. Tate (2006) [63], USA	**Target behavior:** weight loss through calorie restriction and increased physical activity<br>**Target group:** overweight adults (N=192) aged 20 to 65 with a BMI of 27 to 40, willing to use meal replacements	**a. IBC:** 1. feedback on performance; 2. self-monitoring; 3. feedback on progress<br>**IE:** -<br>**PS:** e-buddy network system, message board<br>**CS:** -<br>**EP:** 2 weekly emails with prompts, behavioral lessons and weight loss tips<br>**UD, II:** -	**Login:** median 20 logins on both public and study website (median 2 logins to public website)<br>**IBC:** avg. 11.4 ± 9.2 online diary submissions
		**b. IBC:** 1. self-monitoring<br>**IE:** -<br>**PS:** e-buddy network system, message board<br>**CS:** counselor support containing feedback on progress through weekly e-mails<br>**EP:** 2 weekly emails with prompts, a behavioral lesson and weight loss tips, weekly e-mail from counselor with feedback on performance, progress and overcoming barriers, motivation and answers to questions<br>**UD, II:** -	**Login:** median 32.5 logins on both public and study website (median 9 logins to public website)<br>**IBC:** avg. 17.2 ± 8.7 online diary submissions
		**c. IBC:** 1. self-monitoring; 2. feedback on progress<br>**IE:** -<br>**PS:** e-buddy network system<br>**CS:** -<br>**EP:** weekly emails with prompts and weight loss tips<br>**UD, II:** -	**Login:** median 34 logins on both public and study website (median 20 logins to public website)
28. Webber (2008) [64], USA	**Target behavior:** weight loss through physical activity and dietary habits<br>**Target group:** adult women (N=66) aged 22 to 65 with a BMI of 25 to 40	**a. IBC:** 1. self-monitoring<br>**IE:** website links<br>**PS:** message board<br>**CS:** counselor support through weekly moderated online chat group sessions<br>**EP:** -<br>**UD:** weekly lessons<br>**II:** -	[d]**Login:** avg. 42.8 logins<br>**IBC:** avg. 7.5 completed weekly self-monitoring diaries<br>**PS:** avg. 2.4 postings on message board<br>**CS:** avg. 8 attended chat sessions
		**b. IBC:** 1. self-monitoring<br>**IE:** website links<br>**PS:** message board<br>**CS, EP:** -<br>**UD:** weekly lessons<br>**II:** -	[d]**Login:** avg. 39.7 logins<br>**IBC:** avg. 9.1 completed weekly self-monitoring diaries<br>**PS:** avg. 7.2 postings on message board
29. Van Wier (2009) [65], Netherlands	**Target behavior:** weight loss through sustainable lifestyle changes (reduction of calories through fat, sugar and alcohol and increasing physical activity)<br>**Target group:** employees (N=1386) aged 18 years and older with a BMI of 25 or higher	**IBC, IE, PS:** -<br>**CS:** counselor support through e-mail by commenting on homework assignments and answering of additional questions<br>**EP:** counselor e-mail with intervention content, e-mail/phone prompt max twice a week by not logging on<br>**UD:** weekly modules<br>**II:** -	**Access program content:** 86%<br>[d]**Completion first visit:** 74% completed at least first module<br>**CS:** median 5 counseled sessions [IQR 1 to 10]

30. Wing (2006) [66], USA	**Target behavior:** weight gain prevention with emphasis on daily self-weighing and self-regulation<br>**Target group:** adults (N=314) with a loss of at least 10% of their body weight during prior 2 years	**IBC:** 1. self-monitoring<br>**IE:** -<br>**PS:** message board,<br>**CS:** counselor-led weekly chat sessions (1st month), monthly chat sessions (2-18 months), counselor support through e-mail depending on weight gain during program<br>**EP:** e-mail with intervention content in case of weight gain during program<br>**UD:** weekly tips<br>**II:** small gifts by maintaining weight	**IBC:** reporting weight 82%/baseline to 6 months, 69%/7-12 months, 55%/13-18 months<br>**CS:** attendance chat room sessions 66%/ baseline to 6 months, 41%/7-12 months), 34%/13-18 months)
**D. Smoking cessation**			
31. Balmford (2008) [67], Australia	**Target behavior:** smoking cessation<br>**Target group:** general smoking population (N=23,656)	**IBC:** 1.feedback on cognitive and behavioral processes; 2. feedback on progress<br>**IE, PS, CS:** -<br>**EP:** e-mail prompts<br>**UD, II:** -	[d]**Revisit website:** 27%; revisit before prompt 20%; revisit after prompt 80%
32. Brendryen (2008) [68], Norway	**Target behavior:** smoking cessation<br>**Target group:** adults (N=290) aged 18 and older, willing to quit without NRT	**IBC:** 1. action planning; 2. self-monitoring<br>**IE, PS, CS:** -<br>**EP:** e-mail prompts, mobile phone text messages and voice response messages (reactive log-on calls), and post-quitting, support phone calls (proactive log-off calls)<br>**UD:** daily, during first phase of intervention<br>**II:** -	**Landing website:** 0.3% out of 947,059 times the banner was displayed<br>[d]**Login:** avg. 26 logins ± 13 (59%) [range 0-44]<br>[d]**Completion whole intervention:** 60%<br>**EP:** avg. 26 ± 16 (62%) log-on calls [range 0-42]; avg. 53 ± 37 (52%) log-off calls [range 0-102]
33. Brendryen (2008) [69], Norway	**Target behavior:** smoking cessation<br>**Target group:** adults (N=396) aged 18 and older, smoking 10 or more cigarettes daily who were willing to quit	**IBC:** 1. action planning; 2. self-monitoring<br>**IE, PS, CS:** -<br>**EP:** e-mail prompts, mobile phone text messages and voice response messages (reactive log-on calls), and post-quitting, support phone calls (proactive log-off calls)<br>**UD:** daily, during first phase of intervention<br>**II:** -	[d]**Login:** avg. 30 logins ± 13 (68%) [range 0-44]<br>[d]**Completion whole intervention:** 77%<br>**EP:** avg. 30 ± 16 (71%) log-on calls [range 0-42]; avg. 69 ± 35 (66%) log-off calls [range 0-104]
34. Cobb (2005) [70], USA	**Target behavior:** smoking cessation<br>**Target group:** adult smokers (N=1501)	**IBC:** 1. feedback on cognitive and behavioral processes; 2. setting quit date; 3. self-monitoring<br>**IE:** money and ‘life saved’ calculator, real time notification of forum/chat/internal e-mail messages, identification of quitting buddies, and searchable list of smoking cessation resources<br>**PS:** forums, internal e-mail system, chat rooms, and buddy system<br>**CS:** individual counseling support by online counselors, and ask the expert in online forum<br>**EP:** tailored e-mail support messages<br>**UD, II:** -	**Duration visit:** quitters median 12 min per session [range 7-20]; smokers median 14.5 min [range 8-23]; quitters median 103 min total online [range 33-339]; smokers median 33 min [range 17-82.5]<br>**Pages visited:** quitters median 128 pages [range 31-366]; smokers median 34 pages [range 17-87]<br>**Login:** quitters median 9 logins [range 1-42]; smokers median 2 [range 1-5]<br>[d]**Revisit website:** 53%<br>**PS:** 19% quitters and 5% smokers posted on forum; 19% quitters and 10% smokers with at least one buddy; 25% quitters and 9% smokers sent e-mail to at least one person; 41% quitters and 21% smokers received e-mail from at least one person

35. Danaher (2006) [32], USA	**Target behavior:** cessation of smokeless tobacco<br>**Target group:** adult smokeless tobacco users (N=2375)	**a. IBC:** 1. feedback on cognitive and behavioral processes; 2. planning to quit<br>**IE:** video based testimonials, and website links<br>**PS:** support forum<br>**CS:** ask the expert forum<br>**EP:** e-mails prompts, and support e-mails<br>**UD:** new information in stay quit part<br>**II:** -	**Access program content:** 96%<br>**Visit duration:** median overall 28.99 min<br>[d]**Completion first visit:** 64% continued on day of enrolment<br>**Login:** median 2 logins<br>**IBC:** 63% setting quit date<br>**IE:** 18% used outside links; 68% video testimonial<br>**PS:** 38% posted message<br>**CS:** 5% posted message
		**b. IBC: -**<br>**IE:** website links<br>**PS, CS, EP, UD, II: -**	**Access program content:** 93%<br>**Visit duration:** median overall 12.50 min<br>[d]**Completion first visit:** 39% continued on day of enrolment<br>**Login:** median 1 logins<br>**IE:** 32% used outside links
36. Feil (2003) [71], USA	**Target behavior:** smoking cessation<br>**Target group:** adults (N=606) aged 18 and older in at least contemplator stage of quitting	**IBC:** 1. planning to quit<br>**IE:** anti-tobacco entertainment, e.g., puzzles and video's, and website links<br>**PS:** bulletin board, and chat room<br>**CS:** ask the expert<br>**EP:** e-mail messages as intervention component<br>**UD, II:** -	[d]**Login:** avg. 7.9 logins ± 38.8; women 8.3 logins ± 39.2; men 6.7 logins ± 41.5<br>**IBC:** 63% accessed personalized quit-plan segment<br>**PS:** avg. 3.7 posting ± 30.3; women 4.4 postings ± 34.6; men 1.8 postings ± 14.2
37. Graham (2007) [72], USA	**Target behavior:** smoking cessation<br>**Target group:** smoking employees of a multinational information technology company (N=1776)	**IBC:** 1. feedback on cognitive and behavioral processes; 2. setting quit date; 3. self-monitoring<br>**IE:** money and 'life saved' calculator, real time notification of forum/chat/internal e-mail messages, identification of quitting buddies, and searchable list of smoking cessation resources<br>**PS:** forums, internal e-mail system, chat rooms, and buddy system<br>**CS:** individual counseling support by online counselors, and ask the expert in online forum<br>**EP:** tailored e-mail support messages<br>**UD:** -<br>**II:** benefits premium discount of $11 per month to a maximum of $132 for the year, for use of intervention	[d]**Visit duration:** avg. 15 min per visit ± 10.3 min, median 12 min; avg. 205 min in total ± 2161, median 23 min<br>[d]**Pages visited:** avg. 95 pages ± 518; median of 18 pages<br>[d]**Login:** avg. 12 logins ± 89.7; median 2 logins [range 0-1846]<br>[d]**Revisit:** 53%; never accessed program <1%, 1 time 46%, 2 times 19%; 3 times 10%, 4 or more times 24%<br>**IBC:** 62% used expert system for quit date; 18% used medication expert system<br>**PS:** 7% quitters and 0.4% continued smokers posted on forum; 8% quitters and 4% continued smokers had a buddy; 9% quitters and 2% continued smokers belonged to club; 12% quitters and 6% continued smokers sent e-mail to other members
38. Houston (2008) [73], USA	**Target behavior:** smoking cessation<br>**Target group:** current smokers (N=231)	**a. IBC:** 1. feedback on cognitive constructs; 2. planning to quit<br>**IE:** small games and quizzes, decisional balance calculator, and library<br>**PS:** forum<br>**CS:** ask the expert<br>**EP, UD, II:** -	**Duration visit:** median 18 min; <3 min 24%, 3-10 min 13%, >10-25 min 31%, >25 min 32%<br>***Remark:*** *16% of both phases used forum; 4% of both phases used ask the expert; 1% (3 participants)j of both phases revisited the website*<br>**IBC:** 69% used self-management strategies; 50% used family help module; 56% used talking to your doctor module
		**b. IBC:** 1. feedback on cognitive constructs; 2. planning to quit<br>**IE:** small games and quizzes, decisional balance calculator, and library<br>**PS:** forum<br>**CS:** ask the expert<br>**EP, UD, II:** -	**Duration visit:** median 8 min; <3 min 31%, 3-10 min 30%, >10-25 min 15%, >25 min 23%<br>**IBC:** 58% used self-management strategies; 29% used family help module; 33% used talking to your doctor module
39. Lenert (2003) [22], USA	**Target behavior:** smoking cessation<br>**Target group:** smokers (N=49) who had completed a previous web-based survey on cessation needs and who had failed to quit but were ready to quit	**IBC:** 1. feedback on behavior; 2. self-monitoring<br>**IE, PS, CS:** -<br>**EP:** e-mail prompts<br>**UD, II:** -	**Access program content:** 86%<br>[d]**Completing first visit:** 25% completed all modules; avg. 2 of 8 modules were completed<br>**Login:** median 2 logins<br>**IBC:** 82% set quit date

40. McKay (2008) [74], USA	**Target behavior:** a. smoking cessation; b. smoking cessation, through improving physical activity<br>**Target group:** current smokers (N=2328) aged 18 and older interested in quitting in next 30 days and willingness to engage in moderate PA	**a. IBC:** 1. feedback on cognitive and behavioral processes; 2. planning to quit<br>**IE:** -<br>**PS:** forum<br>**CS:** ask the expert forum<br>**EP, UD, II:** -	[d]**Duration visit:** avg. 8.4 min per visit; total avg. 18.04 min ± 22.18<br>[d]**Login:** avg. 2.14 logins ± 3.66
		**b. IBC:** 1. feedback on performance; 2. goal setting; 3. action planning; 4. self-monitoring; 5. feedback on progress<br>**IE:** -<br>**PS:** forum<br>**CS, EP, UD, II:** -	[d]**Duration visit:** avg. 8.1 min per visit; total avg. time 14.02 min ± 17.09<br>[d]**Login:** avg. 1.74 logins ± 2.43
41. Saul (2007) [75], USA	**Target behavior:** smoking cessation<br>**Target group:** adult (N=607) aged 18 and over who accessed the site as a current smoker and had not already quit at the time of registration	**IBC:** 1. feedback on cognitive constructs; 2. setting quit date; 3. self-monitoring<br>**IE:** money and 'life saved' calculator, real time notification of forum/chat/internal e-mail messages, identification of quitting buddies, and searchable list of smoking cessation resources<br>**PS:** forums, internal e-mail system, chat rooms, and buddy system<br>**CS:** individual counseling support by online counselors, ask the expert in online forum<br>**EP:** tailored e-mail support messages<br>**UD, II:** -	**Login:** in past 6 month never logged in: 52%; 1-3 logins: 29%; 4 or more logins: 19%<br>[d]**Revisit website:** 48%; 32% of non-responders and 53% of responders
42. Severson (2008) [31], USA	**Target behavior:** smoke free tobacco cessation<br>**Target group:** adult smokeless tobacco users (N=2523) aged 18 and over who were thinking of quitting	**a. IBC:** 1. feedback on cognitive and behavioral processes; 2. planning to quit<br>**IE:** video's and website links<br>**PS:** forum<br>**CS:** ask the expert forum<br>**EP:** e-mail support and e-mail prompts<br>**UD:** new information in stay quit part<br>**II:** -	**Access program content:** 95%<br>[d]**Duration visit:** avg. 11.1 min per visit; total avg. 37.51 min; median 27.59 min [range 0.01-439]<br>[d]**Login:** avg. 3.39 logins; median 2 logins [range 1-37]<br>**PS:** avg. 2.91 posts on forum; median 1 post [range 1-106]<br>**CS:** avg. 0.33 posts on expert forum; median 1 post [range 1-17]
		**b. IBC:** -<br>**IE:** website links and FAQ<br>**PS, CS, EP, UD, II:** -	**Access program content:** 93%<br>[d]**Duration visit:** avg. 8.3 min per visit; total avg. 15.77 min; median 11.62 min [range 0.04-186]<br>[d]**Login:** avg. 1.9 logins; median 1 logins [range 1-25]
43. Stoddard (2005) [76], USA	**Target behavior:** smoking cessation<br>**Target group:** smokers (N=538) aged 18 and over who smoked 1 or more cigarettes daily	**IBC:** 1. feedback on nicotine dependency and depressive symptoms<br>**IE:** website links<br>**PS, CS, EP, UD, II:** -	**Landing website:** 0.5% of direct mail<br>[d]**Completion first visit:** 91%
44. Stoddard (2008) [77], USA	**Target behavior:** smoking cessation<br>**Target group:** adult federal employees and contractors (N=1375) aged 18 and over who were willing to quit smoking	**a. IBC:** 1. feedback on cognitive processes<br>**IE:** -<br>**PS:** bulletin board/forum<br>**CS:** online counseling<br>**EP:** e-mail support and prompt messages<br>**UD, II:** -	[d]**Duration visit:** avg. 18.0 min<br>**IBC:** hits various tools varied from 242 to 437 hits<br>**PS:** 12% used bulletin board

		**b. IBC:** 1. feedback on cognitive processes<br>**IE, PS:** -<br>**CS:** online counseling<br>**EP:** e-mail support and prompt messages<br>**UD, II:** -	[d]**Duration visit:** avg. 11.1 min<br>**IBC:** hits various tools varied from 240 to 413 hits
45. Strecher (2005) [78], England and Ireland	**Target behavior:** smoking cessation among nicotine patch users<br>**Target group:** adult smokers (N=3971) aged 18 and older smoking more than 10 cigarettes a day, who purchased nicotine patches and target quit date within 7 days	**a. IBC:** 1. feedback on cognitive and behavioral processes<br>**IE, PS, CS:**<br>**EP:** e-mail support messages<br>**UD:** three sequential newsletters via website<br>**II:** -	**Access program content:** 88%
		**b. IBC, IE, PS, CS:** -<br>**EP:** e-mail support messages<br>**UD, II:** -	**Access program content:** 88%
46. Strecher (2008) [79, 80], USA	**Target behavior:** smoking cessation<br>**Target group:** adult smokers (N=944) aged 21-70, smoked at least 100 cigarettes in lifetime and currently smoking at least 10 cigarettes a day, who were seriously considering quitting in next 30 days	**a. IBC:** 1. feedback on cognitive and behavioral processes, and on barrier identification and solutions; 2. setting quit date<br>**IE:** success stories<br>**PS, CS:** -<br>**EP:** e-mail prompts<br>**UD:** weekly new opened sections<br>**II:** -	**IBC:** avg. 2.6 opened sections
		**b. IBC:** 1. feedback on cognitive and behavioral processes, and on barrier identification and solutions; 2. setting quit date<br>**IE:** success stories<br>**PS, CS, EP, UD, II:** -	**IBC:** avg. 3.1 opened sections
47. Swartz. (2006) [81], USA	**Target behavior:** smoking cessation<br>**Target group:** smokers (N=351) aged 18 and older, currently smoking daily, willing to make quit attempt in the next 30 days	**IBC:** 1. feedback on cognitive and behavioral processes and barrier identification; 2. planning to quit<br>**IE:** video segments, and audio segments in combination with animated graphics<br>**PS, CS, EP, UD, II:** -	**IBC:** 56% viewed quit plan module and set actual quit date; 49% viewed overcoming barriers; 42% viewed voiding situations that prompt cravings; 42% viewed dealing with cravings; 35% viewed benefits of quitting smoking; 70% viewed at least one optional section within modules
48. Wang (2004) [82], Switzerland	**Target behavior:** smoking cessation<br>**Target group:** smokers in general (N=18,361)	**IBC:** 1. feedback on cognitive and behavioral processes; 2. feedback on progress<br>**IE:** -<br>**PS:** forum<br>**CS:** -<br>**EP:** bi-monthly e-mail prompts<br>**UD, II:** -	[d]**Revisit website:** 20%
**E. Alcohol reduction**			
49. Cloud (2001) [83], USA	**Target behavior:** abstaining or controlled drinking<br>**Target group:** adult problem drinkers (N=2813) aged 18 and older	**IBC:** 1. feedback on performance and cognitive constructs<br>**IE:** FAQ and website links<br>**PS, CS, EP, UD, II:** -	**Landing website:** 10,253 hits during 172 study period<br>**Access program content:** 27%<br>[d]**Pages visited:** avg. 1.4 additional web pages

50. Cunningham (2000) [84], Canada	**Target behavior:** problem drinking, drinking habits<br>**Target group:** NR (N=214)	**IBC:** 1. feedback on performance<br>**IE, PS, CS, EP, UD, II:** -	**Landing website:** more than 500 hits each month<br>**Accessed program content:** 14%<br>[d]**Completion first visit:** 88%
51. Lieberman (2006) [85], USA	**Target behavior:** alcohol abuse<br>**Target group:** adults (N=288)	**a. IBC:** 1. feedback on cognitive constructs<br>**IE:** online guide<br>**PS, CS, EP, UD, II:** -	**Access program content:** 89%<br>[d]**Completion first visit:** 90% completed all 4 modules; 0% 1 module; 0% 2 modules; 10% 3 modules
		**b. IBC:** 1. feedback on cognitive constructs<br>**IE, PS, CS, EP, UD, II:** -	[d]**Completion first visit:** 83% completed all 4 modules; 5% 1 module; 4% 2 modules; 9% 3 modules
52. Linke (2004) [86], Linke (2005) [87], UK	**Target behavior:** excessive alcohol consumption<br>**Target group:** adults (N=1319) with a FAST score of 3 of above	**IBC:** 1. feedback on performance and cognitive constructs; 2. self-monitoring<br>**IE:** quizzes, recreational area, blood alcohol concentration calculator, FAQ about heavy drinking, mouse-overs<br>**PS:** discussion group<br>**CS:** -<br>**EP:** e-mail/SMS as intervention content, e-mail as prompt<br>**UD:** new consecutive intervention modules<br>**II:** -	**Landing website:** 7,581 hits during 6-month study period<br>**Accessed program content:** 17%<br>[d]**Completion first visit:** 62%<br>[d]**Completion whole intervention:** 6% completed all 6 modules (1 module 62%, 2-5 modules 32%, 3 modules 20%, 4 modules 14%, 5 modules 10%)
53. Linke (2007)[88], UK	**Target behavior:** promotion of sensible drinking<br>**Target group:** adults (N=10,000) with a FAST score of 3 of above	**IBC:** 1. feedback on performance and cognitive constructs; 2. self-monitoring<br>**IE:** quizzes, recreational area, blood alcohol concentration calculator, FAQ about heavy drinking, mouse-overs<br>**PS:** discussion group<br>**CS:** -<br>**E-mail/Phone:** e-mail/SMS as intervention content, e-mail as prompt<br>**Update:** new consecutive intervention modules<br>**II:** -	[d]**Completion first visit:** 89%<br>[d]**Completion whole intervention:** 17% completed all 6 weeks (89% week 1, 40% week 2, 30% week 3, 24% week4, 19% week 5)
54. Matano (2007) [89], USA	**Target behavior:** reduction of alcohol consumption<br>**Target group:** employees at a worksite (N=229) with low or moderate risk for alcohol-related problems	**a. IBC:** 1. feedback on performance, stress levels, and cognitive constructs; 2. self-monitoring; 3. feedback on progress<br>**IE:** mini-workshop, animations, website links<br>**PS:** forum<br>**CS, EP, UD, II:** -	**Access program content:** 72%<br>[d]**Duration visit:** avg. 16.7 min ± 12.3 for moderate-risk, avg. 19.7 min ± 16.0 for low-risk<br>[d]**Login:** avg. 1.3 logins ± 0.5 for moderate-risk, avg. 1.3 logins ± 0.5 for low-risk
		**b. IBC:** 1. feedback on stress levels and cognitive constructs; 2. self-monitoring; 3. feedback on progress<br>**IE:** mini-workshop, animations, website links<br>**PS:** forum<br>**SC, EP, UD, II:** -	[d]**Duration visit:** avg. 18.9 min ± 16.4 for moderate-risk, avg. 16.8 min ± 12.9 for low-risk<br>[d]**Login:** avg. 1.4 logins ± 0.9 for moderate-risk, avg. 1.4 logins ± 0.9 for low-risk
55. Riper (2008) [90], Netherlands	**Target behavior:** reduction of alcohol consumption<br>**Target group:** excessive and hazardous drinkers (N=261) aged 28-65 without professional help	**a. IBC:** 1. feedback on behavior and cognitive constructs; 2. goal setting; 3. self-monitoring; 4. feedback on progress<br>**IE:** animations, website links<br>**PS:** discussion forum<br>**CS, EP:** -<br>**Update:** tip of the day<br>**II:** -	**Access program content:** 45%
		**b. IBC, IE, PS, CS, EP, UD, II:** -	**Access program content:** 51%
56. Saitz (2004) [91], USA	**Target behavior:** Alcohol use<br>**Target group:** adult website visitors (N=39,842) aged 18 and above who complete screening about their own drinking	**IBC:** feedback on performance<br>**IE:** online information library, searchable national database, website links<br>**PS, CS, EP, UD, II:** -	[d]**Duration visit:** average of 5.25 min<br>**IBC:** after receiving results 19% chose the 'Learn More' or 'Get Help' option

57. Westrup (2003) [92], USA	**Target behavior:** reduction of alcohol consumption<br>**Target group:** highly educated workforce (N=187)	**a. IBC:** 1. feedback performance, stress levels and cognitive constructs; 2. self-monitoring; 3. feedback on progress<br>**IE:** mini-workshop, animations, website links<br>**PS:** forum<br>**CS, EP, UD, II:** -	***Remark:*** *no distinction was made between Internet interventions regarding visit duration and login*<br>**Duration visit:** avg. 19.9 min ± 14.2 [range 3-68]; high risk avg. 19.9 min ± 13.3, moderate risk avg. 14.6 min ± 10.8, low risk avg. 14.8 ± 10.7)<br>**Login:** 1 login 78%, 2 logins 16%, 3 logins 4%, 4 or 5 logins 3%
		**b. IBC:** 1. feedback on stress levels and cognitive constructs: 2. self-monitoring; 3. feedback on progress<br>**IE:** mini-workshop, animations, website links<br>**PS:** forum<br>**CS, EP, UD, II:** -	
**F. Combination of behaviors**			
58. Cook (2007) [93], USA	**Target behavior:** nutrition/weight management, fitness/physical activity, and stress management<br>**Target group:** employees of human resources company (N=419)	**IBC:** 1. feedback on performance and cognitive constructs; 2. goal setting; 3. action planning; 4. self-monitoring; 5. feedback on progress<br>**IE:** BMI and target heart rate calculator, video testimonials and skills training, interactive exercises and videos, FAQ, website links<br>**PS, CS, EP, UD, II:** -	**Access program content:** 10%<br>**IBC:** 14% never used nutrition module, 45% one time, 25% twice, 15% more than twice; 20% never used physical activity module, 55% one time, 16% twice, 9% more than twice
59. Cowdery (2007) [94], USA	**Target behavior:** Smoking cessation, weight management, nutrition, physical activity, alcohol, injury prevention, mental health, skin protection<br>**Target group:** university staff (N=90)	**IBC:** 1. Feedback on performance and cognitive constructs<br>**IE:** website links<br>**PS, CS, EP, UD, II:** -	[d]**Completing first visit:** 13% of eligible participants
60. Oenema (2008) [95], Netherlands	**Target behavior:** saturated fat intake, physical activity, smoking cessation<br>**Target group:** adult participants (N=2159) of an online research panel aged 30 and older	**IBC:** 1. feedback on performance and cognitive constructs; 2. action planning; 3. feedback on progress<br>**IE, PS, CS, EP, UD, II:** -	**Access program content:** 81%<br>[d]**Completing first visit:** 93% completed at least one module<br>**IBC:** 72% visited saturated fat module, 72% physical activity module, 60% of the smokers visited smoking module
61. Verheijden (2007) [23], Netherlands	**Target behavior:** health promotion through several lifestyle behaviors aimed at physical activity as core behavior, and dietary habits, alcohol intake, smoking, work, cardio-respiratory fitness, and muscle strength<br>**Target group:** general adult population (N=6272)	**IBC: 1**. feedback on performance; 2. feedback on progress<br>**IE:** self-tests on anthropometrics, cardio-respiratory fitness, and muscle strength<br>**PS, CS:** -<br>**EP:** email as prompt<br>**UD:** availability of follow-up modules<br>**II:** -	[d]**Revisit website:** 10%; 2 times 8%, 3 times 2%, 4 times <1%
62. Ware (2008) [96], UK	**Target behavior:** Weight loss, weight maintenance, physical activity<br>**Target group:** office and manufacturer workers (N=265)	**IBC:** 1. feedback on performance and barrier identification; 2. goal setting; 3. action planning; 4. self-monitoring; 5. feedback on progress<br>**IE:** -<br>**PS:** community message boards, discussion forums<br>**CS:** -<br>**EP:** e-mail and/or mobile phone reminder messages<br>**UD, II:** -	**Access program content:** 88%<br>[d]**Duration visit:** avg. 11.6 min week 1, avg. 8.6 min week 2, avg. 7 min weeks 3-12, resulting in avg. 7.5 min per week<br>[d]**Login:** week 1-2 avg. 6 logins per week, week 3-12 avg. 2 logins per week, resulting in 32 logins during intervention period<br>[d]**Completion whole intervention:** 22%

63. Winett (2007) [97], USA	**Target behavior:** fat, fiber, and fruit and vegetable intake, physical activity<br>**Target group:** adult church members (N=1071)	**a. IBC:** 1. feedback on cognitive constructs; 2. goal setting; 3. self-monitoring; 4. feedback on progress<br>**IE:** audio narrator 'guide', virtual restaurant<br>**PS, CS, EP:** -<br>**UD:** weekly new modules<br>**II:** -	**Access program content:** 80%<br>[d]**Completion whole intervention:** 50%<br>**IBC:** avg. 7.0 modules were viewed; 50% viewed all modules
		**b. IBC:** 1. feedback on cognitive constructs; 2. goal setting; 3. self-monitoring; 4. feedback on progress<br>**IE:** audio narrator 'guide', virtual restaurant<br>**PS, CS, EP:** -<br>**UD:** weekly new modules<br>**II:** -	**Access program content:** 57%<br>[d]**Completion whole intervention:** 25%<br>**IBC:** avg. 4.6 modules were viewed; 25% viewed all modules
64. Woolf (2006) [98], USA	**Target behavior:** healthy diet, physical activity, smoking cessation, and reduced problem drinking<br>**Target group:** adults (N=273) with unhealthy behaviors	**IBC:** 1. feedback on performance and cognitive constructs<br>**IE:** website links, resource library<br>**PS, CS:** -<br>**EP:** e-mail prompts<br>**UD, II:** -	**Landing website:** 932 hits of 25,488 unique patients visited practice during study period<br>**Access program content:** 29%<br>[d]**Completion first visit/intervention:** 94%
		**b. IBC, IE, PS, CS, EP, UD, II: -**	

Notes: NR = not reported; BMI = body mass index; NRT = nicotine replacement therapy; FAST = fast alcohol screening test

[a] Information of publications that evaluated and reported on the same interventions but were separate studies were combined. This applies to the following studies: both studies of Hurling [39, 40], Gold [55] and Micco [60], both studies of Brendryen [68, 69], Cobb [70] with Graham [72] and Saul [75], Danahar [32] and Severson [31], both studies of Linke [86-88], and Matano [89] and Westrup [92]

[b] The main categories of potential exposure promoting elements are abbreviated as: IBC = interactive behavior change strategy; IE = interactive elements; PS = peer support; CS = counselor/therapist support; EP = e-mail and/or phone contact; UD = update intervention website; II = intervention incentive

[c] Both Internet interventions a. from Gold [55] and Micco [60] are identical as they come from the same study but are compared in two publications to a another intervention

[d] Indicate that these exposure measures are incorporated in Table 3 and 4
