# Supplementary material for: Which Intervention Characteristics are Related to More Exposure to Internet-Delivered Healthy Lifestyle Promotion Interventions? A Systematic Review
Source: J Med Internet Res. 2011 Jan 6;13(1):e2. doi: 10.2196/jmir.1639 (PMC3221341; doi:10.2196/jmir.1639)
Supplement: Supplementary file 2 [file jmir_v13i1e2_app2.pdf]

Multimedia Appendix 2 Summary of studies included in this review: target behavior, study characteristics, brief description of Internet intervention content and duration, theory, number of study participants and characteristics of study population

Study[a]	Target behavior	Study characteristics	Brief description of Internet intervention, name and duration	Theory[c]	No. of study participants	Characteristics study population
**A. Physical activity**						
					**Mean:** 6043<br>**Median:** 174	
1. Carr (2008) [36], USA	Physical activity	**Design:** RCT<br>**Recruitment:** advertisements and e-mail solicitation<br>**Conditions:** a: Internet-delivered intervention; b: delayed intent-to-treat control group	**Intervention:** interactive 16-lessons program providing feedback on cognitions and processes of change, goal setting, activity planning and self-monitoring tools, and weekly/bi-weekly e-mail/phone contact with facilitator; an additional print workbook was provided<br>**Name:** Active Living Every Day – Internet (ALED-I)<br>**Duration intervention:** 16 weeks with multiple visits	SCT<br>TTM	32<br>(a: 14; b: 18)	**% female**: 81<br>**Age (SD)**: 45.9 ± 2.7<br>**% > high school:** NR
2. Dunton (2008) [37], USA	Physical activity	**Design:** RCT<br>**Recruitment:** posters, flyers, and e-mails<br>**Conditions**: a. tailored website with weekly e-mails; b. waiting list control group<br>**Incentive:** $25 after completing 4 assessments (baseline, 1, 2, 3 months)	**Intervention:** interactive program providing individualized, stage tailored feedback on performance, cognitions and perceived barriers, 10 weekly follow-up e-mail newsletters<br>**Name:** Women's Fitness Planner<br>**Duration intervention:** accessible during 10 weeks	HBM<br>TTM	156<br>(a: 85; b: 71)	**% female**: 100<br>**Age (SD)**: 42.8 ± 11.6<br>**% > high school:** 97
3. Ferney (2008) [28], Australia	Walking and overall physical activity	**Design:** RCT<br>**Recruitment:** advertisements community newspapers, letterbox drop<br>**Conditions**: a. neighborhood environment-focused website; b. motivational-information website (minimal interactivity)	**a. Intervention:** website providing information about cognitive behavioral strategies in fact sheets, interactive goal setting tool, social support, information on physical activity facilities and walking trails in the local environment and individualized tailored e-mail advice<br>**Name:** Get Up & Go<br>**Duration intervention:** accessible during 26 weeks	EM<br>SCT	106<br>(a: 52; b:54)	**% female**: 72<br>**Age (SD)**: 52.0 ± 4.6<br>**% > high school:** 71
			**b. Intervention:** website with minimal interactivity with 4 main sections based on motivational stages of change and non-tailored e-mail advice<br>**Name:** Active Living Online<br>**Duration intervention:** accessible during 26 weeks	TTM		
4. Herman (2006) [38], USA	Physical activity	**Design:** pretest-posttest<br>**Recruitment:** NR<br>**Condition:** Internet-based program<br>**Incentive:** $150 cash rebate for participating in program	**Intervention:** online program providing goal setting and activity logging, containing feedback on progress and providing e-mail support; in addition participation in in-company sports teams and competitions was promoted<br>**Name:** Virtual Fitness Center Program<br>**Duration intervention:** 12 months with multiple visits	NR	67,324 participants	**% female**: 35<br>**Age (SD)**: 44.0 ± NR<br>**% > high school:** NR

5. Hurling (2007) [39], UK	Total and moderate to vigorous physical activity	**Design:** RCT<br>**Recruitment:** through market research recruitment agency<br>**Conditions:** a. Internet and mobile phone based program combined with wearing physical activity monitors; b. wearing physical activity monitors without feedback and access<br>**Incentive:** £30 for attending screening, £140 for mobile phone costs, and £290 at closeout	**Intervention:** multi-media (website, e-mail and mobile phone) program, providing tailored feedback on behavior, cognitions, and progress, including goal setting, self-monitoring and weekly activity planning tools and option for social support<br>**Name:** Get active!<br>**Duration intervention:** 9 weeks with multiple visits	DB<br>ELM<br>SCPT	77<br>(a: 47; b: 30)	**% female**: 66<br>**Age (SD)**: 40.4 ± 7.6<br>**% > high school:** NR
6. Hurling (2006) [40], UK	Physical activity/ exercise	**Design:** RCT<br>**Recruitment:** advertisement<br>**Conditions:** a. Internet-based exercise motivation and action support system; b: less interactive version of same system; c. reference group with no intervention	**a. Intervention:** support system requiring weekly logins for self-monitoring and activity planning, providing feedback on progress and cognitions, automated dialogue system for barrier identification, option for e-mail or text message reminders of planned activities.<br>**Name:** NR<br>**Duration intervention:** 10 weeks with multiple visits	DB<br>ELM<br>SCPT	66<br>(a. 28; b. 24; c. 14)	**% female**: 74<br>**Age (SD)**: 35 ± NR<br>**% > high school:** NR
			**b. Intervention:** Support system requiring weekly logins for self-monitoring and feedback on progress, less interactive than above<br>**Name:** NR<br>**Duration intervention:** 10 weeks with multiple visits	DB<br>ELM<br>SCPT		
7. Lewis (2008) [41], Marcus (2007) [42], USA	Physical activity and exercise	**Design:** RCT<br>**Recruitment:** newspaper advertisements<br>**Conditions:** a. motivationally tailored Internet intervention; b. 6 researcher selected websites available to the public; c. motivationally tailored print intervention<br>**Incentive:** $10 each month to complete the online questionnaires and activity logs	**a. Intervention:** website providing motivational and educational materials, monthly tailored feedback reports on performance, cognitions and progress, containing goal setting and self-monitoring tools, regular e-mail prompts to revisit the website<br>**Name:** NR<br>**Duration intervention:** 12 months with multiple visits	SCT<br>TTM	249<br>(a. 81; b. 82; c. 86)	**% female**: 83<br>**Age (SD)**: 45.1 ± 9.3<br>**% college graduate:** 67
			**b. Intervention:** website containing 6 links to general physical activity websites and self-monitoring tool; regular e-mail prompts to log onto the website<br>**Name:** NR<br>**Duration intervention:** accessible during 12 months	NR		
8. Leslie (2005) [16], Australia	Physical activity and exercise	**Design:** RCT<br>**Recruitment:** university e-mail list<br>**Conditions:** a. stage-targeted website program; b. stage-targeted print program	**Intervention:** website providing stage-targeted information on cognitive constructs, containing goal setting and activity planning tools; personalized stage-targeted weekly e-mails were used to attract visitors to the website<br>**Name:** Active Living<br>**Duration intervention:** accessible during 8 weeks	TTM	655<br>(a: 327; b: 328)	**% female**: 50<br>**Age (SD)**: 43 ± NR<br>**% secondary school or higher:** 72

9. Plotnikoff (2006) [43], Canada	Raise awareness for physical activity	**Design:** observational<br>**Recruitment:** advertisement cereal package, media, links website, word of mouth (self-reported)<br>**Condition:** Internet-based intervention	**Intervention:** website providing goal setting, self-monitoring (of steps counted through pedometer), activity planning, and containing brief visual feedback on progress<br>**Name:** Canada on the Move<br>**Duration intervention:** NR (one-time users vs. multi-time users)	NR	3175 registrations	**% female**: 77<br>**% Age:** <24: 10.1; 25-44: 46.7; 45-64: 38.5; 65-80+: 4.6<br>**% >- high school:** 98
10. Spittaels (2006) [44], Belgium	Physical activity	**Design:** quasi-experimental<br>**Recruitment:** flyers with and without personal contact<br>**Condition**: interactive tailored Internet program (*remark: intervention a and b are identical, only the way of promotion differed*)	**Intervention:** website providing feedback on performance, perceived barriers and cognitions, including activity planning, and social support<br>**Name:** NR<br>**Duration intervention:** accessible during 2 months with single exposure	SCM<br>TPB	52<br>(a: 46; b: 6)	**% female**: 51<br>**Age (SD)**: 38 ± 11<br>**% > high school:** 66
11. Spittaels (2007) [45], Belgium	Physical activity	**Design:** RCT<br>**Recruitment:** brochures plus e-mail as prompt for first visit<br>**Conditions:** a. Internet advice with repeated feedback; b. Internet advice without repeated feedback; c. waiting list control group	**a. Intervention:** website providing feedback on performance, cognitive constructs and progress, containing activity planning and social support, 7 non-tailored e-mails were used to prompt visits to specific website sections, one to prompt revisiting the intervention for new tailored advice<br>**Name:** NR<br>**Duration intervention:** accessible during 6 months with multiple visits	SCM<br>TPB	434<br>(a: 173; b: 129; c: 132)	**% female**: 66<br>**Age (SD)**: 41.4 ± 5.6<br>**% > high school:** 67
			**b. Intervention:** website providing feedback on performance and cognitive constructs, including activity planning and social support<br>**Name:** NR<br>**Duration intervention:** accessible during 6 months with single exposure	SCM<br>TPB		
12. Steele (2007) [46, 47], Australia	Moderate intensity and lifestyle physical activity	**Design:** RCT<br>**Recruitment:** advertisements local newspapers<br>**Conditions:** a. Internet-only intervention; b. Internet-mediated intervention; c. face-to-face intervention<br>**Incentive:** gift vouchers, water bottles, and sport socks	**a. Intervention:** website consisting of weekly modules aimed at improving self-management skills; program provides feedback on cognitive constructs, goal setting, self-monitoring (using pedometer information), barrier identification, activity planning and including options for online counselor support and 2 face-to-face support sessions<br>**Name:** Health-eSteps (Internet only)<br>**Duration intervention:** 12 weeks with multiple visits	SCT<br>SMM	192<br>(a: 62; b: 65; c: 65)	**% female**: 83<br>**Age (SD)**: 38.7 ± 12.0<br>**% > high school:** NR
			**b. Intervention:** website consisting of weekly modules aimed at improving self-management skills; program provides feedback on cognitive constructs, including goal setting, self-monitoring (using pedometer information), barrier identification, activity planning and including option for online counselor support<br>**Name:** Health-eSteps (Internet with online counselor support)<br>**Duration intervention:** 12 weeks with multiple visits	SCT<br>SMM		

**B. Nutrition**						
					**Mean:** 344<br>**Median:** 285	
13. Buller (2008) [48], Woodall (2007) [49], USA	Fruit and vegetable consumption	**Design:** RCT<br>**Recruitment:** in person by community outreach trainers<br>**Conditions:** a. Internet intervention with immediate access to website; b. control group with delayed access after post-test	**Intervention:** website providing generic information to promote fruit and vegetable intake; e-mail prompts to announce new and updated information<br>**Name:** 5 a day, the Rio Grande Way<br>**Duration intervention:** accessible during 4 month study period with multiple visits	DIT<br>SCT	755<br>(a: 380; b: 375)	**% female**: 88<br>**% age**: <30: 34; 30-39: 17; 40-49: 18; 50-59: 14; >59 14; missing 3<br>**% > high school:** 63
14. Huang (2006) [50], Australia	Purchases of saturated fat	**Design:** RCT<br>**Recruitment:** online pop-up message<br>**Conditions:** a: Internet intervention with tailored advice; b: generic information on static webpage	**a. Intervention:** website providing tailored feedback on selected items with the opportunity to retain or swap purchased item; provided as part of supermarket website<br>**Name:** NR<br>**Duration intervention:** accessible during 5 month study period for multiple shopping episodes	NR	497<br>(a: 251; b: 246)	**% female**: 88<br>**Age (SD)**: 40.0 ± 10<br>**% university:** 62
			**b. Intervention:** website that provided generic non-specific advice about how to choose a diet lower in saturated fat; intervention provided on supermarket website<br>**Name:** NR<br>**Duration intervention:** NR	NR		
15. McNeill (2007) [51], USA	Fruit and vegetable consumption	**Design:** observational<br>**Recruitment:** NR, drawn from sample of larger study of health centers<br>**Condition:** Internet-based intervention	**Intervention:** website providing non-tailored information on overcoming barriers, setting goals, social support and maintaining healthy behavior, supplemented with an e-mail with feedback on intake and suggestions how to increase consumption<br>**Name:** NR<br>**Duration intervention:** accessible during 6 weeks	NR	52 enrolled	**% female**: 73<br>**Age (SD)**: 46 ± 9<br>**% > high school:** 77
16. Papadaki (2005) [52], Papadaki (2006) [53], Scotland	Consumption of four key components of the Mediterranean diet	**Design:** quasi-experimental<br>**Recruitment:** advertisements in newsletters, flyers, postings on Intranet and e-mail advertisements<br>**Conditions:** a. tailored Internet intervention; b. minimal dietary feedback and general healthy-eating brochures	**Intervention:** website providing generic information and recipes, supplemented with tailored feedback letters through e-mail containing feedback on performance, barriers, social cognitions and progress towards goal achievement<br>**Name:** Mediterranean Eating Website<br>**Duration intervention:** 6 months accessible with multiple visits	HBM<br>PAPM<br>SCT<br>TPB<br>TTM	72<br>(a: 53; b. 19)	**% female**: 100<br>**Age (SD)**: 40.5 ± 7.0<br>**% > high school:** 100
**C. Weight management**						
					**Mean:** 1006<br>**Median:** 207	
17. Cussler (2008) [54], USA	Weight maintenance through diet, physical activity and weight gain prevention	**Design:** RCT<br>**Recruitment:** advertisement newspaper and TV<br>**Conditions:** a. Internet intervention; b. self-directed (no intervention) control	**Intervention:** weight maintenance website (after following weight loss intervention) with tools for monitoring progress, peer support and optional counselor support<br>**Name:** NR<br>**Duration intervention:** 12 months with multiple visits	NR	135<br>(a. 66; b. 69)	**% female**: 100<br>**Age (SD)**: 48.2 ± 4.4<br>**% > high school:** NR

18. Glasgow (2007) [21], USA	Weight loss through nutrition and physical activity	**Design:** RCT<br>**Recruitment:** personal letters through medical leaders or notices in general member communications<br>**Conditions:** a. online tailored weight management program with nutrition component and goal setting; b. online tailored weight management program with goal setting; c. online tailored weight management program with; nutrition component; d. online tailored weight management program<br>**Incentive:** US $10 gift certificate by completing follow-up questionnaire	**a. Intervention:** website providing feedback on performance, cognitions and tailored action plans including a goal setting module and additional tailored newsletters regarding nutrition<br>**Name:** Balance Program (goal + nutrition)<br>**Duration intervention a:** 14 weeks with multiple visits	NR	2311<br>(a. 559; b. 584; c. 596; d. 572)	**% female**: 53<br>**% < 60 years:** 53.5<br>**% > high school:** NR
			**b. Intervention:** website providing feedback on performance, cognitions and tailored action plans, including a goal setting module<br>**Name:** Balance Program (goal)<br>**Duration intervention:** 6 weeks with multiple visits	NR		
			**c. Intervention:** website providing feedback on performance, cognitions and tailored action plans with additional tailored newsletters regarding nutrition<br>**Name:** Balance Program (nutrition)<br>**Duration intervention:** 14 weeks with multiple visits	NR		
			**d. Intervention:** website providing feedback on performance, cognitions and tailored action plans<br>**Name:** Balance Program<br>**Duration intervention:** 6 weeks with multiple visits	NR		
19. Gold (2007) [55], USA[b]	Weight loss through reducing calorie intake and increase aerobic activity	**Design:** RCT<br>**Recruitment:** advertisements in local newspaper<br>**Conditions:** a. structured behavioral weight loss program; b. commercial weigh loss website	**a. Intervention:** weekly online lessons with homework assignment and goal setting, providing exercise schedule, facilitator provided weekly/bi-weekly feedback on behavior and self-monitoring, weekly chat sessions with facilitator<br>**Name:** VTrim Program<br>**Duration intervention:** 12 months with multiple visits	NR	124<br>(a. 62; b. 62)	**% female**: 84<br>**Age (SD)**: 47 ± 9<br>**% > high school:** 98
			**b. Intervention:** commercial program consisting of feedback on cognitions, self-monitoring, and feedback on progress, providing peer support and professional online meetings<br>**Name b:** eDiets.com<br>**Duration intervention:** 12 months with multiple visits	NR		
20. Harvey-Berino (2002) [56], USA	Weight loss maintenance through modification of eating and exercise habits	**Design:** RCT<br>**Recruitment:** newspaper advertisements<br>**Conditions:** a. Internet support; b. frequent in-person support; c. minimal in-person support<br>**Incentive:** chance to enter a lottery to win $50 for attending scheduled assessment meetings	**Intervention:** website providing self-monitoring forms accompanied by bi-weekly Internet chat meetings facilitated by group therapist and introduced with short videos; bi-weekly e-mails from group therapist with feedback on behavior and self-monitoring<br>**Name:** NR<br>**Duration intervention:** 12 months with multiple visits	NR	122<br>(a. 40; b. 41; c. 41)	**% female**: 85<br>**Age (SD)**: 48.4 ± 9.6<br>**% > high school:** 90

21. Hunter (2008) [57], USA	Weight gain prevention and weight loss through restricting calorie and fat intake, and increasing physical activity	**Design:** RCT<br>**Recruitment:** e-mail advertisements and flyers<br>**Conditions:** a. behavioral Internet therapy; b. usual care	**Intervention:** Internet-based program containing weekly interactive lessons and self-monitoring tool, feedback from an Internet counselor on performance and progress, including two brief motivational interviewing telephone calls<br>**Name:** Behavioral Internet Therapy (BIT)<br>**Duration intervention:** 24 weeks with multiple visits	MI	446<br>(a. 224; b. 222)	**% female**: 50<br>**Age (SD)**: 33.9 ± 7.3<br>**% high school or some college:** 63
22. McConnon (2007) [58], UK	Weight loss through dietary and physical activity	**Design:** RCT<br>**Recruitment:** posters and flyers in GPs waiting rooms/practices<br>**Conditions:** a. Internet group; b. usual care	**Intervention:** website providing feedback on performance, self-monitoring tool, and feedback on progress, including e-mails prompts for revisiting website<br>**Name:** UK weight control site<br>**Duration intervention:** 12 months with multiple visits	NR	221<br>(a. 111; b. 110)	**% female**: 77<br>**Age (SD)**: 45.8 ± 10.6<br>**% > high school:** NR
23. McCoy (2005) [59], Australia	Weight loss through changing physical activity and dietary behavior	**Design:** Observational<br>**Recruitment:** promotional interviews on radio stations<br>**Condition**: Internet-based program	**Intervention:** Internet service delivering tailored plans for behavior change based on current health status and personal needs and goals with respect to diet and physical activity<br>**Name:** Weight Loss for Diabetes Prevention Program<br>**Duration intervention:** accessible during 10 weeks	NR	808 registrations	**% female**: 75<br>**Age (SD)**: 42.3 ± 11.6<br>**% > high school:** NR
24. Micco (2007) [60], USA[b]	Weight loss through changing eating and exercise behavior	**Design:** RCT<br>**Recruitment:** newspaper advertisements<br>**Conditions:** a. Internet intervention only; b. Internet intervention including in-person support	**a. Intervention:** weekly online lessons with homework assignment and goal setting, providing exercise schedule, facilitator provided weekly/bi-weekly feedback on behavior and self-monitoring, weekly chat sessions with facilitator<br>**Name:** VTrim (Internet only)<br>**Duration intervention:** 12 months with multiple visits	NR	123<br>(a. 62; b. 61)	**% female**: 83<br>**Age (SD)**: 46.7 ± 10.8<br>**% > high school:** 93
			**b. Intervention:** weekly online lessons with homework assignment and goal setting, providing exercise schedule, facilitator provided weekly/bi-weekly feedback on behavior and self-monitoring, monthly in-person group sessions<br>**Name:** VTrim (Internet with in-person support)<br>**Duration intervention:** 12 months with multiple visits	NR		
25. Petersen (2008) [61], USA	Weight management by creating life long habits	**Design:** pretest-posttest<br>**Recruitment:** announcement on employee web portal with limited promotion<br>**Condition:** Internet-based program	**Intervention:** comprehensive, interactive online program providing self-monitoring, and feedback on performance and progress, containing interactive tools and e-mail communications towards personalized goal achievement<br>**Name:** Virtual Food Pro (VFP) program<br>**Duration intervention:** 18 weeks (recommended duration) with multiple visits, however ongoing program	SCM	7743 participants	**% female**: 60<br>**Age (SD)**: NR<br>**% > high school:** NR
26. Tate (2001) [62], USA	Weight loss through calorie restriction and increased physical activity	**Design:** RCT<br>**Recruitment:** series of 2 e-mail messages and an advertisement posted to the work site's Intranet website<br>**Conditions**: a. Internet education + Internet behavior therapy; b. Internet education<br>**Incentive:** $10 and $25 for attending the 3- and 6-month follow-up appointments	**a. Intervention:** website providing a brief review of basic information and organized directory of selected Internet resources (e.g. self-monitoring) and other resources, additionally weekly e-mail lessons and feedback and support from therapist and access to bulletin board<br>**Name:** NR<br>**Duration intervention:** accessible during 24 weeks	NR	91<br>(a: 46; b: 45)	**% female**: 89<br>**Age (SD)**: 40.9 ± 10.6<br>**% > high school:** 91

			**b. Intervention:** website providing a brief review of basic information and organized directory of selected Internet resources (e.g. self-monitoring) and other resources<br>**Name:** NR<br>**Duration intervention:** accessible during 24 weeks	NR		
27. Tate (2006) [63], USA	Weight loss through calorie restriction and increased physical activity	**Design:** RCT<br>**Recruitment:** local newspaper advertisements<br>**Conditions**: a. website with computer-automated e-mail feedback; b. website with human counseling; c. website with no counseling<br>**Incentive:** $25 and $50 for attending the 3- and 6-month follow-up appointments	**a. Intervention:** website with additional study website containing self-monitoring diary and automated feedback on performance and progress, providing social support, and e-mail prompts to complete diary including behavioral lesson<br>**Name:** Slim-Fast Web site combined with website with automated feedback<br>**Duration intervention:** 6 months with multiple visits	CBT	192<br>(a. 61; b. 64; c. 67)	**% female**: 84<br>**Age (SD)**: 49.2 ± 9.8<br>**% > high school:** 55
			**b. Intervention:** website with additional study website containing self-monitoring diary and social support supplemented with human e-mail counseling providing feedback on performance and progress, e-mail prompts to complete diary including behavioral lesson<br>**Name:** Slim-Fast Web site combined with website and human e-mail counseling<br>**Duration intervention:** 6 months with multiple visits	NR		
			**c. Intervention:** website containing self-monitoring tool, providing feedback on progress, including social support and weekly e-mail prompts to report weight<br>**Name:** Slim-Fast Web site<br>**Duration intervention:** accessible during 6 months	NR		
28. Webber (2008) [64], USA	Weight loss through physical activity and dietary habits	**Design:** RCT<br>**Recruitment:** newspaper advertisement<br>**Conditions:** a. intervention website with weekly moderated online chat group sessions; b. intervention website<br>**Incentive:** $40 for attending the follow-up appointment	**a. Intervention:** Internet behavioral program with weekly lessons, self-monitoring, information links and social support supplemented with weekly moderated online chat group sessions<br>**Name:** NR, enhanced website<br>**Duration intervention:** 16 weeks with multiple visits	MI	66<br>(a. 33; b. 33)	**% female**: 100<br>**Age (SD)**: 50.1 ± 9.9<br>**% > high school:** 70
			**b. Intervention:** Internet behavioral program with weekly lessons, self-monitoring, information links and social support<br>**Name:** NR, minimal website<br>**Duration intervention:** 16 weeks with multiple visits	NR		

29. Van Wier (2009) [65], Netherlands	Weight loss through sustainable lifestyle changes (reduction of calories through fat, sugar and alcohol and increasing physical activity)	**Design:** RCT<br>**Recruitment:** health faire, company Intranet and/or personal letter<br>**Conditions:** a. web-based intervention with e-mail counseling; b. intervention materials with phone counseling; c. usual care through lifestyle brochures	**Intervention:** interactive website with 10 homework modules and individualized web pages supplemented with e-mail counseling, also provided with self-help materials<br>**Name:** ALIFE@Work<br>**Duration intervention:** 6 months with multiple visits	SCT	1386<br>(a. 464; b. 462; c. 460)	**% female**: 33<br>**Age (SD)**: 43 ± 8.6<br>**% highly educated:** 60
30. Wing (2006) [66], USA	Weight gain prevention with emphasis on daily self-weighing and self-regulation	**Design:** RCT<br>**Recruitment:** newspaper advertisements, brochures, and contacts with commercial and research weight-control programs<br>**Conditions:** a. Internet-based intervention; b. face-to-face intervention; c. control group receiving quarterly newsletters<br>**Incentive:** $25 for attending the 6- and 12-month assessments and $50 for attending the 18-month assessment	**Intervention:** intervention program with online chat sessions, web-based form for self-monitoring and social support; individual e-mail counseling was offered in case of weight gain until starting weight was reached<br>**Name:** STOP Regain<br>**Duration intervention:** 18 months with multiple visits	SRT	314<br>(a. 104; b. 105; c. 105)	**% female**: 81<br>**Age (SD)**: 51.3 ± 10.1<br>**% > high school:** NR
**D. Smoking cessation**						
					**Mean:** 3437<br>**Median:** 1160	
31. Balmford (2008) [67], Australia	Smoking cessation	**Design:** observational<br>**Recruitment:** promotion through national quit smoking websites<br>**Condition:** tailored automated advice Internet program	**Intervention:** website providing feedback on behavioral strategies, cognitive restructuring, motivation and relapse prevention with the aims to resemble in-persons counseling; website consisted of 5 modules that could be accessed during one visit<br>**Name:** QuitCoach<br>**Duration intervention:** NR, continuously accessible and designed for multiple visits	CBT<br>RPM<br>Perspectives on change (derived from SCM)	23,656 registrations	**% female**: 62<br>**Age (median)**: 24<br>**% > high school:** NR
32. Brendryen (2008) [68], Norway	Smoking cessation	**Design:** RCT<br>**Recruitment:** online banner advertisements on websites or regional newspapers<br>**Conditions:** a. Internet and cell phone based intervention; b. control group receiving 44-page self-help booklet	**Intervention:** multi-media (website, e-mail and SMS text messaging and interactive voice response system (IVR)) program, including a craving helpline and a relapse prevention system with just-in-time therapy; program provides information on a variety of determinants and processes for change, relevant in various phases of the process toward quitting smoking; information is provided through daily websites during the first phase of the intervention, supplemented with text messages and voice response messages<br>**Name:** Happy Ending<br>**Duration intervention:** 54 weeks with multiple visits	BSL<br>CBT<br>SCT<br>SRT	290<br>(a. 144; b. 146)	**% female**: 50<br>**Age (SD)**: 39.6 ± 10.9<br>**% > high school:** 50

33. Brendryen (2008) [69], Norway	Smoking cessation	**Design:** RCT<br>**Recruitment:** Online banner advertisements on Internet newspapers<br>**Conditions:** a. Internet and cell phone –based intervention; b. control group receiving 44-page self-help booklet<br>**Incentive:** free supply of NRT	**Intervention:** multi-media (website, e-mail and SMS text messaging and interactive voice response system (IVR)) program, including a craving helpline and a relapse prevention system with just-in-time therapy; program provides information on a variety of determinants and processes for change, relevant in various phases of the process toward quitting smoking; the information is provided through daily websites during the first phase of the intervention, supplemented with text messages and voice response messages<br>**Name:** Happy Ending<br>**Duration of intervention**: 54 weeks with multiple visits	BSL<br>CBT<br>SCT<br>SRT	396<br>(a. 197; b. 199)	**% female**: 50<br>**Age (SD)**: 36.2 ± 10.2<br>**% > high school:** 41
34. Cobb (2005) [70], USA	Smoking cessation	**Design:** observational<br>**Recruitment:** NR<br>**Conditions:** Internet-based intervention	**Intervention:** website providing feedback on cognitive and behavioral processes, assistance with setting a quit date, action and coping planning, relapse prevention, information about medication support and options for peer and online counseling support.<br>**Name:** QuitNet<br>**Duration of intervention:** NR	SCM	1501	**% female**: 65<br>**Age (SD)**: 35.2 ± 10.8<br>**% > high school:** 80
35. Danaher (2006) [32], USA	Cessation of smokeless tobacco	**Design:** RCT<br>**Recruitment:** print and broadcast media, Google ads, websites links, and mailings to target group, health care and tobacco control professionals<br>**Conditions:** a. enhanced condition web-based program (interactive, tailored, and rich-media program); b. basic condition control website (static, text-based material)	**a. Intervention:** interactive and tailored web-based program offering eight modules, including text-based information (health and behavioral strategies focused on quitting and preventing relapse), video-based testimonials, printable resources, interactive activities, annotated links to other website resources, and two web forums one for support by peers and one for support by experts<br>**Name:** ChewFree.com (enhanced)<br>**Duration intervention:** NR	SCT	2375<br>(a. 1200; b. 1175)	**% female**: NR<br>**Age (SD)**: NR<br>**% > high school:** NR
			**b. Intervention:** website providing a printable self-help smokeless tobacco cessation booklet, printable cessation resources (e.g., describing the use of herbal snuff products, nicotine replacement products), and annotated links to other recommended websites for tobacco cessation<br>**Name:** ChewFree.com (basic)<br>**Duration of intervention:** NR	SCT		

36. Feil (2003) [71], USA	Smoking cessation	**Design:** RCT<br>**Recruitment:** website search engines, banner advertisements, postings to discussion groups, newspaper advertisement and article, brochures at dental clinics and doctor's offices, radio interview<br>**Condition:** Internet-delivered intervention<br>**Incentive:** all subjects who completed the baseline survey were mailed checks for US$10<br>1. email 10 dollar; 2. email 20 dollar, 3 US mail 10 dollar, 4 US mail 20 dollar	**Intervention:** website providing guidance in improving motivation to quit, avoidance and dealing with cravings and setting a quit date, options for social support by peers and experts<br>**Name:** Quit-Smoking-Network<br>**Duration intervention:** NR	NR	606 enrolled	**% female**: 72<br>**% aged 25-54**: 85<br>**% > high school:** 80
37. Graham (2007) [72], USA	Smoking cessation	**Design:** pretest-posttest<br>**Recruitment:** company's Intranet and e-mail<br>**Condition**: Internet-based worksite intervention	**Intervention:** commercial program providing feedback on cognitive and behavioral processes, assistance with setting a quit date, action and coping planning, relapse prevention, information about medication support and unlimited use of options for peer support and online counseling support<br>**Name:** QuitNet<br>**Duration intervention:** NR	SCM	1776 program users	**% female**: 35<br>**Age (SD)**: 44.1 ± 9.6<br>**% > high school:** NR
38. Houston (2008) [73], USA	Smoking cessation	**Design:** quasi-experimental<br>**Recruitment:** Google advertisements<br>**Conditions:** a. website with extensive introduction; b. website with minimal introduction<br>**Incentive:** a $20 gift certificate to a popular online store as reimbursement for completing the questionnaire for both Phase 1 and Phase 2 users	**a. Intervention:** website providing stage tailored self-management advice and advice on how to get assistance and support from others (family, doctors) to quit smoking; elaborate introduction to the program content<br>**Name:** Free2Quit (extensive introduction)<br>**Duration intervention**: NR	HBM<br>SCT<br>TTM	231<br>(a. 105; b. 126)	**% female**: 71<br>**% age**: <30: 19.0; 30-45: 47.6; 46-60: 32.0; >60: 1.3<br>**% > high school:** 63
			**b. Intervention:** website providing stage tailored self-management advice and advice on how to get assistance and support from others (family, doctors) to quit smoking; brief introduction to the program content.<br>**Name:** Free2Quit (minimal introduction)<br>**Duration intervention**: NR	HBM<br>SCT<br>TTM		
39. Lenert (2003) [22], USA	Smoking cessation	**Design:** pretest-posttest<br>**Recruitment:** e-mail invitation<br>**Condition:** web and e-mail based program	**Intervention:** web and e-mail based program primarily aimed at mood management; the program consisted of 8 modules that had to be accessed one at the time but could be completed in one visit, including self-monitoring tools, feedback on behavior, and e-mail prompts for revisits; additional educational information and materials could be browsed<br>**Name:** NR<br>**Duration intervention**: 8 weeks which could be completed in one visit, but also through multiple visits	MM<br>CBT	49 website users	**% female**: 78<br>**Age (SD)**: 46 ± NR<br>**% > high school:** 75

40. McKay (2008) [74], USA	a. smoking cessation, b. smoking cessation through improving physical activity	**Design:** RCT<br>**Recruitment:** advertisements on search engines and website links<br>**Conditions**: a. web-based program with information and behavioral strategies; b. web-based personalized fitness program that would help to quit smoking	**a. Intervention:** website designed to encourage tobacco abstinence via the use of strategies that address each participant's behavior, cognition, environment and self-management skills; provides options for peer and expert support<br>**Name:** Quit smoking network (QSN)<br>**Duration intervention:** NR	SCT	2318<br>(a: 1159; b: 1159)	**% female:** 71<br>**% age:** <30: 22.4; 30-39: 23.4; 40-49: 29.6; >50: 24.6<br>**% > high school:** 68
			**b. Intervention:** website encouraging participants to participate in a fitness program, to help them quit smoking; program provides tailored feedback on performance, providing goal setting, activity planning and monitoring of progress and includes access to additional online resources and a web-forum for peer support.<br>**Name:** Active Lives<br>**Duration intervention:** NR	SCT		
41. Saul (2007) [75], USA	Smoking cessation	**Design:** pretest-posttest<br>**Recruitment:** additional screen during registration process<br>**Condition:** Internet-based intervention<br>**Incentive:** a US $10 check for completing follow-up survey	**Intervention:** Internet-based program providing feedback on cognitive and behavioral processes, assistance with setting a quit date, action and coping planning, relapse prevention, information about medication support and options for peer support and online counseling support<br>**Name:** Quitplan.com<br>**Duration intervention:** NR	SCM	607 registered visitors	**% female:** 64<br>**Age (SD):** 37.9 ± NR<br>**% > high school:** 82
42. Severson (2008) [31], USA	Smoke free tobacco cessation	**Design:** RCT<br>**Recruitment:** print and broadcast media, Google ads, websites links, and mailings to target group, health care and tobacco control professionals<br>**Conditions:** a. interactive, tailored web-based program (enhanced condition); b. more linear, text-based website (basic condition)<br>**Incentive:** $10 for each follow-up assessment and an additional $20 by completing all three follow-up assessments	**a. Intervention:** web-based intervention, providing tailored feedback on cognitive and behavioral processes for change, guidance in making a quit plan, support in staying quit, and options for support by peers and counselor<br>**Name:** ChewFree.com (enhanced condition)<br>**Duration intervention:** NR, but multiple visits	SCT	2523<br>(a. 1260; b. 1263)	**% female:** 2<br>**Age (SD):** 36.8 ± 9.6<br>**% > high school:** 81
			**b. Intervention:** website providing linear text-based printable information on quitting and useful resources<br>**Name:** ChewFree.com (basic condition)<br>**Duration intervention:** NR	SCT		
43. Stoddard (2005) [76], USA	Smoking cessation	**Design:** pretest-posttest<br>**Recruitment:** announcements on list serves, registration with popular search engines, and a direct e-mail<br>**Conditions:** Internet self-help educational intervention	**Intervention:** website providing tailored feedback on nicotine dependency and depressive symptoms and subsequent access to an online quit smoking and nicotine replacement guide.<br>**Name:** NR<br>**Duration intervention:** one time visit	NR	538 participants	**% female:** 74<br>**% age:** 18-24: 5.2; 25-44: 48.5; 45-64: 42.7; 65+: 3.5<br>**% > high school:** 54

44. Stoddard (2008) [77], USA	Smoking cessation	**Design:** RCT<br>**Recruitment:** e-mail invitations<br>**Conditions:** a. website including a bulletin board; b. same website without bulletin board<br>**Incentive:** either a 100 minute prepaid calling or US$7.40 postal stamp for those not completing follow-up after	**a. Intervention:** website providing online quit guide, targeted to stages of change, 5 unique (downloadable) self-help materials targeted to specific groups, evidence based information on positive health changes after quitting, option to contact counselor for support and peer support through bulletin board<br>**Name:** Smokefree.gov (including bulletin board)<br>**Duration intervention:** NR	NR	1375<br>(a. 684; b. 691)	**% female:** 54<br>**Age (SD):** 43.6 ± 10.3<br>**% > high school:** 87
			**b. Intervention:** website providing online quit guide, targeted to stages of change, 5 unique (downloadable) self-help materials targeted to specific groups, evidence based information on positive health changes after quitting, option to contact counselor for support<br>**Name:** Smokefree.gov (without bulletin board)<br>**Duration intervention:** NR	NR		
45. Strecher (2005) [78], England and Ireland	Smoking cessation among nicotine patch users	**Design:** RCT<br>**Recruitment:** advertisements on nicotine patches<br>**Conditions:** a. tailored intervention website; b. non-tailored web-based smoking cessation materials<br>**Incentive:** £5 of oral care products by completing either 6- or 12-week, by completing £20 of oral care products	**a. Intervention:** provision of a web-based cessation guide and three sequential online tailored newsletters providing feedback on cognitive and behavioral concepts and behavioral support messages delivered via email; opportunity to identify a supportive person who subsequently received an e-mail message with tailored advice<br>**Name:** CQ plan<br>**Duration of intervention:** 10 weeks with multiple visits	CBM of smoking cessation<br>RPM	3971<br>(a. 1991; b. 1980)	**% female:** 57<br>**Age (SD):** 36.9 ± 10.2<br>**% > high school:** NR
			**b. Intervention:** website providing non-tailored information on cognitive-behavioral concepts<br>**Name:** NR<br>**Duration intervention:** accessible during 10 weeks	CBM of smoking cessation<br>RPM		
46. Strecher (2008) [79, 80], USA	Smoking cessation	**Design:** RCT<br>**Recruitment:** invitation letter to smokers selected by 2 health care organizations<br>**Conditions:** a. intervention website with multiple visits; b. intervention website with single visits<br>**Incentive:** 10-week course of NRT	**a. Intervention:** website consisting of 5 modules that could only be accessed through multiple visits, providing tailored feedback on cognitive and behavioral processes, feedback on barrier identification, and possibility to set quit date, containing success stories and e-mail prompt to encourage revisits<br>**Name:** Project Quit (multiple visits)<br>**Duration intervention:** 5 weeks with multiple visits	CBM of smoking cessation<br>RPM	944<br>(a. 487; b. 457)	**% female:** 60<br>**Age (SD):** 46.3 ± NR<br>**% > high school:** 64
			**b. Intervention:** website consisting of 5 modules that could be accessed in a single visit, providing tailored feedback on cognitive and behavioral processes, feedback on barrier identification, and possibility to set quit date, containing success stories<br>**Name:** Project Quit (single visit)<br>**Duration intervention:** 1 time visit	CBM of smoking cessation<br>RPM		

47. Swartz (2006) [81], USA	Smoking cessation	**Design:** RCT<br>**Recruitment:** through large worksites (posters, brochures, link Intranet, e-mail employees, electronic newsletters)<br>**Conditions:** a. Internet treatment condition; b. wait list control group	**Intervention:** website providing tailored feedback on cognitive and behavioral constructs and on planning to quit, containing video and audio fragments; separate content is provided for users from different race/ethnicity, sex and age<br>**Name:** 1-2-3 Smokefree<br>**Duration intervention:** one time visit, but multiple visits was encouraged	NR	351<br>(a. 171; b. 180)	**% female:** 52<br>**% age:** 18-25: 7.4: 26-39: 38.2; 40-55: 48.4; > 55: 6.0<br>**% > high school:** NR
48. Wang (2004) [82], Switzerland	Smoking cessation	**Design:** observational study<br>**Recruitment:** via links and search engines<br>**Condition:** web-based intervention	**Intervention:** online feedback program, providing tailored counseling letters with feedback on cognitive and behavioral constructs and feedback on progress, option to access forum and additional documents<br>**Name:** Stop-Tabac<br>**Duration intervention:** NR	RPM<br>TPB<br>TTM	18,361 unique users	**% female:** 49<br>**Age (SD):** 36 ± 11<br>**Average years of schooling:** 15
**E. Alcohol reduction**						
					**Mean:** 6128<br>**Median:** 288	
49. Cloud (2001) [83], USA	Abstaining or controlled drinking	**Design:** observational<br>**Recruitment:** e-mail posting in newsgroup and search engines<br>**Condition:** interactive web-based intervention	**Intervention:** website providing feedback on performance, risk for addiction, and cognitions, with access to online self-help manual<br>**Name:** Carebetter.com<br>**Duration intervention:** one time visit during 172 trial period	NR	2813 registrations	**% female**: 36<br>**Age (SD)**: 32 ± 10.3<br>**Education in years (SD):** 14.8 ± 3.6
50. Cunningham (2000) [84], Canada	Problem drinking, drinking habits	**Design:** observational study<br>**Recruitment:** NR<br>**Condition:** tailored Internet program	**Intervention:** website providing feedback on performance, normative feedback, and educational information<br>**Name:** Try our free drinking evaluation<br>**Duration intervention:** one time visit	NR	214 registrations	**% female**: 58<br>**Age (SD)**: 33.8 ± 12.6<br>**% > high school:** NR
51. Lieberman (2006) [85], USA	Alcohol abuse	**Design:** RCT<br>**Recruitment:** no advertisement but spontaneously through search engines<br>**Conditions:** a. intervention with feedback in multimedia context<br>b. intervention with feedback in html text form	**a. Intervention:** multimedia website providing feedback on the negative effects of alcohol consumption on every day life, including an online guide who leads the visitor through the feedback process<br>**Name:** Alcohol Checkup (multimedia context)<br>**Duration intervention:** one time visit during 18 month	NR	288<br>(a. NR; b. NR)	**% female**: NR<br>**Age (SD)**: NR<br>**% > high school:** NR
			**b. Intervention:** website providing feedback on performance in a html text form<br>**Name:** Alcohol Checkup (html text format)<br>**Duration intervention:** one time visit during 18 months	NR		
52. Linke (2004) [86], Linke (2005) [87],UK	Excessive alcohol consumption	**Design:** observational study<br>**Recruitment:** press releases and news items in national media, 50,000 leaflets distributed to GPs, articles in professional publications in alcohol field, links in search engines and on appropriate health websites<br>**Condition**: Internet-based interactive intervention	**Intervention:** website providing feedback on performance and cognitive constructs, self-monitoring and social support, providing educational materials; program consists of 6 consecutive intervention modules designed to be accessed at weekly intervals<br>**Name:** Down Your Drink (DYD)<br>**Duration intervention:** 6 weeks with multiple visits	CBT<br>MET<br>RPM<br>SCM	1319 registrations	**% female**: 44<br>**Age (SD)**: NR<br>**% > high school:** NR

53. Linke (2007) [88], UK	Promotion of sensible drinking	**Design:** observational study<br>**Recruitment:** Off-line advertising campaign<br>**Condition:** Internet-based interactive intervention	**Intervention:** website providing feedback on performance and cognitive constructs, self-monitoring and social support, providing educational materials; program consists of 6 consecutive intervention modules designed to be accessed at weekly intervals<br>**Name:** Down Your Drink (DYD)<br>**Duration intervention:** 6 weeks with multiple visits	CBT<br>MET<br>RPM<br>SCM	10,000 registrations	**% female**: 51<br>**Age (SD)**: 37.4 ± 9.8<br>**% > high school:** NR
54. Matano (2007) [89], USA	Reduction of alcohol consumption	**Design:** RCT<br>**Recruitment:** mailing of a descriptive recruitment flyer<br>**Conditions**: a. intervention with full individualized feedback; b. intervention with limited individualized feedback<br>**Incentive:** $20 + t-shirt	**a. Intervention:** website providing tailored feedback on performance, stress levels, cognitions and progress, self-monitoring and social support, provision of mini-workshops<br>**Name:** Coping Matters (full individualized feedback)<br>**Duration intervention:** accessible during 90 days	SLT	229<br>(a. NR; b. NR)	**% female**: 78<br>**Age (SD)**: 39.9 ± 11.3<br>**% > high school:** 83.9
			**b. Intervention:** website providing generic information on alcohol consumption and tailored feedback on stress levels and cognitions, however limited compared to above<br>**Name:** Coping Matters (limited individualized feedback)<br>**Duration intervention:** accessible during 90 days	SLT		
55. Riper (2008) [90], Netherlands	Reduction of alcohol consumption	**Design:** RCT<br>**Recruitment:** advertisements in national newspapers and on health related websites<br>**Conditions:** a. web-based interactive self-help intervention; b. online psycho-educational brochure	**a. Intervention:** web-based self-help program providing feedback on behavior, cognitive constructs and progress, providing goal setting, self-monitoring, and social support<br>**Name:** Minder Drinken (Drinking Less)<br>**Duration intervention:** 6 weeks recommended treatment period with multiple visits	Cognitive behavioral and self-control principles	261<br>(a. 130; b. 131)	**% female**: 49<br>**Age (SD)**: 46 ± 9.0<br>**% vocational and academic education:** 69.7
			**b. Intervention:** website that provided a web-based psycho-educational brochure on the effects of alcohol use on physical and social functioning.<br>**Name:** NR<br>**Duration intervention:** 1 time visit	NR		
56. Saitz (2004) [91], USA	Alcohol use	**Design:** Observational study<br>**Recruitment:** national banner-ad public service campaign on hundreds of commercial websites, features as a resource on large television program and linked to online stories, distribution of flyers, referrals from Internet search engines<br>**Condition:** web-based intervention	**Intervention:** website providing feedback on performance, and access to additional information through online information library and searchable national database<br>**Name:** Alcohol screening<br>**Duration intervention:** one visit during 14-month period	HBM	39,842 registrations	**% female**: 33<br>**Age (SD)**: 32 ± 11<br>**% > high school:** NR
57. Westrup (2003) [92], USA	Reduction of alcohol consumption	**Design:** quasi-experimental<br>**Recruitment:** brochure mailings<br>**Conditions:** a. full individualized feedback; b. limited individualized feedback	**a. Intervention:** website providing tailored feedback on performance, stress levels, cognitions and progress, providing self-monitoring and social support, provision of mini-workshops<br>**Name:** Coping Matters (full individualized feedback)<br>**Duration intervention:** NR, with multiple visits	SLT	187<br>(a. NR; b. NR)	**% female**: 77<br>**Age (SD)**: 40.9 ± 11.5<br>**% > high school:** 81

			**b. Intervention:** website providing generic information on alcohol consumption and tailored feedback on stress levels and cognitions, however limited compared to above<br>**Name:** Coping Matters (limited individualized feedback)<br>**Duration intervention:** NR, with multiple visits	SLT		
**F. Combination of behaviors**						
					**Mean:** 1499<br>**Median:** 419	
58. Cook (2007) [93], USA	Nutrition/weight management, fitness/physical activity, and stress management	**Design:** RCT<br>**Recruitment:** e-mail letter with online flyer from management, and posters<br>**Conditions:** a: web-based condition; b: print materials<br>**Incentive:** monetary incentives of $50/survey and raffle prize of $500	**Intervention:** multimedia (website, video) multi-component program, providing feedback on dietary and physical activity behaviors, cognitions and progress, including interactive calorie logbook, goal setting and action planning options, video testimonials, skills training and audio narration; intervention consists of three extensive programs for improving diet and physical activity and reduce stress, each taking 2-3 hours to complete.<br>**Name:** Health Connection<br>**Duration intervention:** NR, multiple visits were encouraged	SCT<br>TTM	419<br>(a: 209; b: 210)	**% female**: 72<br>**Age (SD)**: 42 ± NR<br>**% > high school:** 95
59. Cowdery (2007) [94], USA	Smoking cessation, weight management, nutrition, physical activity, alcohol, injury prevention, mental health, skin protection	**Design:** observational study<br>**Recruitment:** print and electronic communication<br>**Condition:** web-based tailored program	**Intervention:** commercial, health risk assessment program providing feedback on performance and stage of change relevant cognitions, information on associated risk factors and action planning tool<br>**Name:** NR<br>**Duration intervention:** 1 visit during 4-month implementation period	HBM<br>SCT<br>TTM	90 participants	**% female**: 82<br>**Age (SD)**: 45 ± NR<br>**% > high school:** 94
60. Oenema (2008) [95], Netherlands	Saturated fat intake, physical activity, and smoking cessation	**Design:** RCT<br>**Recruitment:** e-mail<br>**Conditions:** a. tailored Internet intervention; b. no intervention waiting list control group<br>**Incentive:** €10 for completing study	**Intervention:** website providing tailored feedback on behavior, cognitions, and progress (for smoking cessation only), providing formation of implementation intentions<br>**Name:** Gezondlevencheck (Healthy Life Check)<br>**Duration intervention:** 1 visit	PAPM<br>SCSM (modified version of SCM)	2159<br>(a: 1080; b. 1079)	**% female**: 54<br>**Age (SD)**: 43.6 ± 10.1<br>**% medium or high educational:** 73
61. Verheijden (2007) [23], Netherlands	Health promotion through several lifestyle behaviors aimed at physical activity as core behavior, and dietary habits, alcohol intake, smoking, work, cardio-respiratory fitness, and muscle strength	**Design:** observational study<br>**Recruitment:** press release and free publicity in newspapers and magazines<br>**Condition:** web-based program	**Intervention:** online health promotion program providing feedback on performance and progress, containing self-tests; program consists of different modules that could be accessed by revisits<br>**Name:** Dutch National Health Test<br>**Duration intervention:** 2 weeks with multiple visits	NR	6272 visitors	**% female**: 66<br>**Age (SD)**: 36 ± 13<br>**% intermediate to (very) high educational level:** 90

62. Ware (2008) [96], UK	Weight loss, weight management, physical activity	**Design: o**bservational study<br>**Recruitment:** leaflet distribution during working hours<br>**Conditions**: web-based and monitoring device-based program	**Intervention:** multi-media (Internet, e-mail and mobile phone) program providing tailored feedback on performance and progress and goal setting, action planning, and social support; self-monitoring data is collected through accelerometers and weighing scales connected with the program through Bluetooth<br>**Name:** MiLife<br>**Duration intervention:** 12 weeks with multiple visits	SCPT<br>DB	265 visitors	**% female**: 51<br>**Age (SD)**: 40.9 ± 8.1<br>**% > high school:** NR
63. Winett (2007) [97], USA	Fat, fiber, and fruit and vegetable intake, physical activity	**Design:** RCT<br>**Recruitment:** pulpit announcements, flyers, posters, bulletins and kickoff luncheons<br>**Conditions:** a. Internet program with series of church-based support; b. Internet program; c. waiting list control group<br>**Incentive:** $20 for assessments at pretest, $30 at posttest, and $40 at follow-up	**a. Intervention:** website providing tailored feedback on performance and progress, goal setting and self-monitoring, audio narrated; use of the program was promoted by prompts from the pulpit and in in-church bulletins and newsletters; newsletters also provided feedback on church wide achievement of nutrition and physical activity goals<br>**Name:** Guide to Health (with church-based support)<br>**Duration intervention:** 12 weeks with multiple visits	SCT	1071<br>(a. 364; b. 364; c. 343)	**% female**: 67<br>**Age (SD)**: 51.4 ± 15.8<br>**% > high school:** NR
			**Intervention:** website providing tailored feedback on performance and progress, goal setting and self-monitoring, audio narrated; use of the program was promoted by prompts from the pulpit and in in-church bulletins and newsletters; without feedback on church wide achievement of nutrition and physical activity goals<br>**Name:** Guide to Health (Internet only)<br>**Duration intervention:** 12 weeks with multiple visits	SCT		
64. Woolf (2006) [98], USA	Healthy diet, physical activity, smoking cessation, and reduced problem drinking	**Design:** quasi-experimental<br>**Recruitment:** aggressive promotion by physicians and nurses of primary care practices, wall papers, and telephone hold-line messages<br>**Conditions:** a. intervention website with tailored health advice; b. control group directed to static pages with limited information	**a. Intervention:** website providing tailored feedback on performance and cognitions, including access to tailored resource library with website links to local and national organizations and agencies<br>**Name:** My Healthy Living (tailored advice)<br>**Duration intervention:** 1 visit during 9 month period	TTM	273<br>(a. 177; b. 96)	**% female + age**: a. 79% female and <50; b. 71% female and <50<br>**% > high school:** NR
			**b. Intervention:** website providing static generic information pages on the 4 health behaviors, and general health promotion tips<br>**Name:** My Healthy Living (static)<br>**Duration intervention:** 1 visit during 9 month period	NR		

Notes: NR = not reported; RCT = randomized controlled trial; NRT = nicotine replacement therapy

[a] Information of publications that evaluated and reported on the same interventions but were separate studies were combined. This applies to the following studies: both studies of Hurling [39, 40], Gold [55] and Micco [60], both studies of Brendryen [68, 69], Cobb [70] with Graham [72] and Saul [75], Danahar [32] and Severson [31], both studies of Linke [86-88], and Matano [89] and Westrup [92]

[b] Both Internet interventions a. from Gold [55] and Micco [60] are identical as they come from the same study but are compared in two publications to a another intervention

[c] BSL = Behavioral Skills Learning; CBM = Cognitive–Behavioral Methods; CBT = Cognitive Behavioral Therapy; DB = Decisional Balance; DIT = Diffusion of Innovations Theory; ELM = Elaboration Likelihood Model; EM = Ecological Models; HBM = Health Belief Model; MET = Motivational Enhancement Therapy; MI = Motivational Interviewing; MM = Mood Management; PAPM = Precaution Adoption Process Model; RPM = Relapse Prevention Model; SCM = Stage of Change Model; SCSM = Social Cognitive Stages Model; SCPT = Social Comparison Theory; SCT = Social Cognitive Theory; SLT = Social Learning Theory; SMM = Self-Management Model; SRT = Self-Regulation Theory; TPB = Theory of Planned Behavior; TTM = Transtheoretical Model
